# Accelerating Medicines Partnership® Schizophrenia (AMP® SCZ): Rationale and Study Design of the Largest Global Prospective Cohort Study of Clinical High Risk for Psychosis

**DOI:** 10.1093/schbul/sbae011

**Published:** 2024-03-07

**Authors:** Cassandra M J Wannan, Barnaby Nelson, Jean Addington, Kelly Allott, Alan Anticevic, Celso Arango, Justin T Baker, Carrie E Bearden, Tashrif Billah, Sylvain Bouix, Matthew R Broome, Kate Buccilli, Kristin S Cadenhead, Monica E Calkins, Tyrone D Cannon, Guillermo Cecci, Eric Yu Hai Chen, Kang Ik K Cho, Jimmy Choi, Scott R Clark, Michael J Coleman, Philippe Conus, Cheryl M Corcoran, Barbara A Cornblatt, Covadonga M Diaz-Caneja, Dominic Dwyer, Bjørn H Ebdrup, Lauren M Ellman, Paolo Fusar-Poli, Liliana Galindo, Pablo A Gaspar, Carla Gerber, Louise Birkedal Glenthøj, Robert Glynn, Michael P Harms, Leslie E Horton, René S Kahn, Joseph Kambeitz, Lana Kambeitz-Ilankovic, John M Kane, Tina Kapur, Matcheri S Keshavan, Sung-Wan Kim, Nikolaos Koutsouleris, Marek Kubicki, Jun Soo Kwon, Kerstin Langbein, Kathryn E Lewandowski, Gregory A Light, Daniel Mamah, Patricia J Marcy, Daniel H Mathalon, Patrick D McGorry, Vijay A Mittal, Merete Nordentoft, Angela Nunez, Ofer Pasternak, Godfrey D Pearlson, Jesus Perez, Diana O Perkins, Albert R Powers, David R Roalf, Fred W Sabb, Jason Schiffman, Jai L Shah, Stefan Smesny, Jessica Spark, William S Stone, Gregory P Strauss, Zailyn Tamayo, John Torous, Rachel Upthegrove, Mark Vangel, Swapna Verma, Jijun Wang, Inge Winter-van Rossum, Daniel H Wolf, Phillip Wolff, Stephen J Wood, Alison R Yung, Carla Agurto, Mario Alvarez-Jimenez, Paul Amminger, Marco Armando, Ameneh Asgari-Targhi, John Cahill, Ricardo E Carrión, Eduardo Castro, Suheyla Cetin-Karayumak, M Mallar Chakravarty, Youngsun T Cho, David Cotter, Simon D’Alfonso, Michaela Ennis, Shreyas Fadnavis, Clara Fonteneau, Caroline Gao, Tina Gupta, Raquel E Gur, Ruben C Gur, Holly K Hamilton, Gil D Hoftman, Grace R Jacobs, Johanna Jarcho, Jie Lisa Ji, Christian G Kohler, Paris Alexandros Lalousis, Suzie Lavoie, Martin Lepage, Einat Liebenthal, Josh Mervis, Vishnu Murty, Spero C Nicholas, Lipeng Ning, Nora Penzel, Russell Poldrack, Pablo Polosecki, Danielle N Pratt, Rachel Rabin, Habiballah Rahimi Eichi, Yogesh Rathi, Avraham Reichenberg, Jenna Reinen, Jack Rogers, Bernalyn Ruiz-Yu, Isabelle Scott, Johanna Seitz-Holland, Vinod H Srihari, Agrima Srivastava, Andrew Thompson, Bruce I Turetsky, Barbara C Walsh, Thomas Whitford, Johanna T W Wigman, Beier Yao, Hok Pan Yuen, Uzair Ahmed, Andrew (Jin Soo) Byun, Yoonho Chung, Kim Do, Larry Hendricks, Kevin Huynh, Clark Jeffries, Erlend Lane, Carsten Langholm, Eric Lin, Valentina Mantua, Gennarina Santorelli, Kosha Ruparel, Eirini Zoupou, Tatiana Adasme, Lauren Addamo, Laura Adery, Munaza Ali, Andrea Auther, Samantha Aversa, Seon-Hwa Baek, Kelly Bates, Alyssa Bathery, Johanna M M Bayer, Rebecca Beedham, Zarina Bilgrami, Sonia Birch, Ilaria Bonoldi, Owen Borders, Renato Borgatti, Lisa Brown, Alejandro Bruna, Holly Carrington, Rolando I Castillo-Passi, Justine Chen, Nicholas Cheng, Ann Ee Ching, Chloe Clifford, Beau-Luke Colton, Pamela Contreras, Sebastián Corral, Stefano Damiani, Monica Done, Andrés Estradé, Brandon Asika Etuka, Melanie Formica, Rachel Furlan, Mia Geljic, Carmela Germano, Ruth Getachew, Mathias Goncalves, Anastasia Haidar, Jessica Hartmann, Anna Jo, Omar John, Sarah Kerins, Melissa Kerr, Irena Kesselring, Honey Kim, Nicholas Kim, Kyle Kinney, Marija Krcmar, Elana Kotler, Melanie Lafanechere, Clarice Lee, Joshua Llerena, Christopher Markiewicz, Priya Matnejl, Alejandro Maturana, Aissata Mavambu, Rocío Mayol-Troncoso, Amelia McDonnell, Alessia McGowan, Danielle McLaughlin, Rebecca McIlhenny, Brittany McQueen, Yohannes Mebrahtu, Martina Mensi, Christy Lai Ming Hui, Yi Nam Suen, Stephanie Ming Yin Wong, Neal Morrell, Mariam Omar, Alice Partridge, Christina Phassouliotis, Anna Pichiecchio, Pierluigi Politi, Christian Porter, Umberto Provenzani, Nicholas Prunier, Jasmine Raj, Susan Ray, Victoria Rayner, Manuel Reyes, Kate Reynolds, Sage Rush, Cesar Salinas, Jashmina Shetty, Callum Snowball, Sophie Tod, Gabriel Turra-Fariña, Daniela Valle, Simone Veale, Sarah Whitson, Alana Wickham, Sarah Youn, Francisco Zamorano, Elissa Zavaglia, Jamie Zinberg, Scott W Woods, Martha E Shenton

**Affiliations:** Centre for Youth Mental Health, The University of Melbourne, Parkville, VIC, Australia; Orygen, Parkville, VIC, Australia; Centre for Youth Mental Health, The University of Melbourne, Parkville, VIC, Australia; Orygen, Parkville, VIC, Australia; Department of Psychiatry, Hotchkiss Brain Institute, University of Calgary, Calgary, AB, Canada; Centre for Youth Mental Health, The University of Melbourne, Parkville, VIC, Australia; Orygen, Parkville, VIC, Australia; Department of Psychiatry, Yale University School of Medicine, New Haven, CT, USA; Connecticut Mental Health Center, New Haven, CT, USA; Department of Child and Adolescent Psychiatry, Institute of Psychiatry and Mental Health, Hospital General Universitario Gregorio Marañón, IiSGM, CIBERSAM, Instituto de Salud Carlos III, School of Medicine, Universidad Complutense, Madrid, Spain; Department of Psychiatry, McLean Hospital and Harvard Medical School, Boston, MA, USA; Department of Psychiatry, Semel Institute for Neuroscience and Human Behavior, University of California, Los Angeles, Los Angeles, CA, USA; Department Biobehavioral Sciences and Psychology, Semel Institute for Neuroscience and Human Behavior, University of California, Los Angeles, Los Angeles, CA, USA; Department of Psychiatry, Brigham and Women’s Hospital and Harvard Medical School, Boston, MA, USA; Department of Software Engineering and Information Technology, École de technologie supérieure, Montréal, Canada; School of Psychology, Institute for Mental Health, University of Birmingham, Birmingham, UK; Early Intervention for Psychosis Services, Birmingham Women’s and Children’s NHS Foundation Trust, Birmingham, UK; Centre for Youth Mental Health, The University of Melbourne, Parkville, VIC, Australia; Orygen, Parkville, VIC, Australia; Department of Psychiatry, University of California, San Diego, CA, USA; Department of Psychiatry, Perelman School of Medicine, University of Pennsylvania, Philadelphia, PA, USA; Department of Psychiatry, Yale University School of Medicine, New Haven, CT, USA; Department of Psychology, Yale University, New Haven, CT, USA; IBM T.J. Watson Research Center, Yorktown Heights, NY, USA; Department of Psychiatry, University of Hong Kong, Pok Fu Lam, Hong Kong; Department Biobehavioral Sciences and Psychology, Semel Institute for Neuroscience and Human Behavior, University of California, Los Angeles, Los Angeles, CA, USA; Olin Neuropsychiatry Research Center, Hartford Hospital, Hartford, CT, USA; Discipline of Psychiatry, University of Adelaide, Adelaide, SA, Australia; Basil Hetzel Institute, Woodville, SA, Australia; Department Biobehavioral Sciences and Psychology, Semel Institute for Neuroscience and Human Behavior, University of California, Los Angeles, Los Angeles, CA, USA; General Psychiatry Service, Treatment and Early Intervention in Psychosis Program (TIPP–Lausanne), Lausanne University Hospital and University of Lausanne, Lausanne, Switzerland; Department of Psychiatry, Icahn School of Medicine at Mount Sinai, New York, NY, USA; Department of Psychiatry, Donald and Barbara Zucker School of Medicine at Hofstra/Northwell, Hempstead, NY, USA; Institute of Behavioral Science, Feinstein Institutes for Medical Research, Northwell Health, Manhasset, NY, USA; Department of Child and Adolescent Psychiatry, Institute of Psychiatry and Mental Health, Hospital General Universitario Gregorio Marañón, IiSGM, CIBERSAM, Instituto de Salud Carlos III, School of Medicine, Universidad Complutense, Madrid, Spain; Centre for Youth Mental Health, The University of Melbourne, Parkville, VIC, Australia; Orygen, Parkville, VIC, Australia; Centre for Neuropsychiatric Schizophrenia Research, CNSR Mental Health Centre, Glostrup, Copenhagen, Denmark; Department of Psychology and Neuroscience, Temple University, Philadelphia, PA, USA; Department of Psychosis Studies, King’s College London, London, UK; Department of Brain and Behavioral Sciences, University of Pavia, Pavia, Italy; Cambridgeshire and Peterborough NHS Foundation Trust, Cambridge, UK; Department of Psychiatry, University of Cambridge, Cambridge, UK; Department of Psychiatry, IMHAY, University of Chile, Santiago, Chile; Behavioral Health Services, PeaceHealth Medical Group, Eugene, OR, USA; Copenhagen Research Centre for Mental Health, Mental Health Copenhagen, University of Copenhagen, Denmark; Department of Psychology, University of Copenhagen, Copenhagen, Denmark; Department of Medicine, Brigham and Women’s Hospital, Harvard Medical School, Boston, MA, USA; Department of Biostatistics, Harvard T.H. Chan School of Public Health and Harvard Medical School, Boston, MA, USA; Department of Psychiatry, Washington University School of Medicine, St. Louis, MO, USA; Department of Psychiatry, University of Pittsburgh School of Medicine, Pittsburgh, PA, USA; Department of Psychiatry, Icahn School of Medicine at Mount Sinai, New York, NY, USA; Department of Psychiatry, Faculty of Medicine and University Hospital Cologne, University of Cologne, Cologne, Germany; Department of Psychiatry, Faculty of Medicine and University Hospital Cologne, University of Cologne, Cologne, Germany; Department of Psychiatry, Donald and Barbara Zucker School of Medicine at Hofstra/Northwell, Hempstead, NY, USA; Institute of Behavioral Science, Feinstein Institutes for Medical Research, Northwell Health, Manhasset, NY, USA; Department of Radiology, Brigham and Women’s Hospital, Boston, MA, USA; Department of Psychiatry, Beth Israel Deaconess Medical Center and Harvard Medical School, Boston, MA, USA; Department of Psychiatry, Chonnam National University Medical School, Gwangju, Korea; Mindlink, Gwangju Bukgu Mental Health Center, Gwangju, Korea; Department of Psychiatry and Psychotherapy, Ludwig Maximilian University of Munich, Munich, Germany; Department Biobehavioral Sciences and Psychology, Semel Institute for Neuroscience and Human Behavior, University of California, Los Angeles, Los Angeles, CA, USA; Department of Psychiatry, Massachusetts General Hospital and Harvard Medical School, Boston, MA, USA; Department of Psychiatry, Seoul National University College of Medicine, Seoul, Korea; Department of Neuropsychiatry, Seoul National University Hospital, Seoul, Korea; Department of Psychiatry and Psychotherapy, Jena University Hospital, Jena, Germany; Department of Psychiatry, McLean Hospital and Harvard Medical School, Boston, MA, USA; Department of Psychiatry, University of California, San Diego, CA, USA; Veterans Affairs San Diego Health Care System, San Diego, CA, USA; Department of Psychiatry, Washington University Medical School, St. Louis, MO, USA; Northwell Health, Glen Oaks, NY, USA; Department of Psychiatry and Behavioral Sciences and Weill Institute for Neurosciences, University of California, San Francisco, San Francisco, CA, USA; Mental Health Service 116D, Veterans Affairs San Francisco Health Care System, San Francisco, CA, USA; Centre for Youth Mental Health, The University of Melbourne, Parkville, VIC, Australia; Orygen, Parkville, VIC, Australia; Department of Psychology, Northwestern University, Evanston, IL, USA; Copenhagen Research Centre for Mental Health, Mental Health Copenhagen, University of Copenhagen, Denmark; Department of Clinical Medicine, University of Copenhagen, Copenhagen, Denmark; Department of Psychiatry, Yale University School of Medicine, New Haven, CT, USA; Connecticut Mental Health Center, New Haven, CT, USA; Department Biobehavioral Sciences and Psychology, Semel Institute for Neuroscience and Human Behavior, University of California, Los Angeles, Los Angeles, CA, USA; Department of Psychiatry, Massachusetts General Hospital and Harvard Medical School, Boston, MA, USA; Department of Psychiatry, Yale University School of Medicine, New Haven, CT, USA; Olin Neuropsychiatry Research Center, Hartford Hospital, Hartford, CT, USA; CAMEO, Early Intervention in Psychosis Service, Cambridgeshire and Peterborough NHS Foundation Trust, Cambridge, UK; Department of Medicine, Institute of Biomedical Research (IBSAL), Universidad de Salamanca, Salamanca, Spain; Department of Psychiatry, University of North Carolina at Chapel Hill, Chapel Hill, NC, USA; Department of Psychiatry, Yale University School of Medicine, New Haven, CT, USA; Connecticut Mental Health Center, New Haven, CT, USA; Department of Psychiatry, Perelman School of Medicine, University of Pennsylvania, Philadelphia, PA, USA; Prevention Science Institute, University of Oregon, Eugene, OR, USA; Department of Psychological Science, University of California, Irvine, Irvine, CA, USA; PEPP-Montreal, Douglas Research Centre, Montreal, Quebec, Canada; Department of Psychiatry, McGill University, Montreal, Quebec, Canada; Department of Psychiatry and Psychotherapy, Jena University Hospital, Jena, Germany; Centre for Youth Mental Health, The University of Melbourne, Parkville, VIC, Australia; Orygen, Parkville, VIC, Australia; Department of Psychiatry, Beth Israel Deaconess Medical Center and Harvard Medical School, Boston, MA, USA; Department of Psychology, University of Georgia, Athens, GA, USA; Department of Psychiatry, Yale University School of Medicine, New Haven, CT, USA; Connecticut Mental Health Center, New Haven, CT, USA; Department of Psychiatry, Chonnam National University Medical School, Gwangju, Korea; Department of Software Engineering and Information Technology, École de technologie supérieure, Montréal, Canada; Birmingham Womens and Childrens, NHS Foundation Trust, Birmingham, UK; Department of Radiology, Massachusetts General Hospital and Harvard Medical School, Boston, MA, USA; Department of Psychosis, Institute of Mental Health, Singapore; Shanghai Mental Health Center, Shanghai Jiaotong University School of Medicine, Shanghai, China; Institute of Behavioral Science, Feinstein Institutes for Medical Research, Northwell Health, Manhasset, NY, USA; Department of Psychiatry, Perelman School of Medicine, University of Pennsylvania, Philadelphia, PA, USA; Department of Psychology, Emory University, Atlanta, GA, USA; Centre for Youth Mental Health, The University of Melbourne, Parkville, VIC, Australia; Orygen, Parkville, VIC, Australia; School of Psychology, University of Birmingham, Edgbaston, UK; Institute of Mental and Physical Health and Clinical Translation (IMPACT), Deakin University, Geelong, VIC, Australia; School of Health Sciences, University of Manchester, Manchester, UK; IBM T.J. Watson Research Center, Yorktown Heights, NY, USA; Centre for Youth Mental Health, The University of Melbourne, Parkville, VIC, Australia; Orygen, Parkville, VIC, Australia; Centre for Youth Mental Health, The University of Melbourne, Parkville, VIC, Australia; Orygen, Parkville, VIC, Australia; Youth Early Detection/Intervention in Psychosis Platform (Plateforme ERA), Service of Child and Adolescent Psychiatry, Department of Psychiatry, Lausanne University Hospital and The University of Lausanne, Lausanne, Switzerland; Department of Radiology, Brigham and Women’s Hospital, Boston, MA, USA; Department of Psychiatry, Yale University School of Medicine, New Haven, CT, USA; Connecticut Mental Health Center, New Haven, CT, USA; Department of Psychiatry, Donald and Barbara Zucker School of Medicine at Hofstra/Northwell, Hempstead, NY, USA; Institute of Behavioral Science, Feinstein Institutes for Medical Research, Northwell Health, Manhasset, NY, USA; IBM T.J. Watson Research Center, Yorktown Heights, NY, USA; Department Biobehavioral Sciences and Psychology, Semel Institute for Neuroscience and Human Behavior, University of California, Los Angeles, Los Angeles, CA, USA; Department of Psychiatry, McGill University, Montreal, Quebec, Canada; Department of Psychiatry, Yale University School of Medicine, New Haven, CT, USA; Connecticut Mental Health Center, New Haven, CT, USA; Department Psychiatry, Beaumont Hospital, Dublin 9, Ireland; Department of Psychiatry, Royal College of Surgeons in Ireland, Dublin 2, Ireland; School of Computing and Information Systems, The University of Melbourne, Parkville, VIC, Australia; Department of Psychiatry, McLean Hospital and Harvard Medical School, Boston, MA, USA; Department Biobehavioral Sciences and Psychology, Semel Institute for Neuroscience and Human Behavior, University of California, Los Angeles, Los Angeles, CA, USA; Department of Psychiatry, Yale University School of Medicine, New Haven, CT, USA; Connecticut Mental Health Center, New Haven, CT, USA; Centre for Youth Mental Health, The University of Melbourne, Parkville, VIC, Australia; Orygen, Parkville, VIC, Australia; Department of Psychiatry, University of Pittsburgh School of Medicine, Pittsburgh, PA, USA; Department of Psychiatry, Perelman School of Medicine, University of Pennsylvania, Philadelphia, PA, USA; Department of Psychiatry, Perelman School of Medicine, University of Pennsylvania, Philadelphia, PA, USA; Department of Psychiatry, Brigham and Women’s Hospital and Harvard Medical School, Boston, MA, USA; Department of Psychiatry, Semel Institute for Neuroscience and Human Behavior, University of California, Los Angeles, Los Angeles, CA, USA; Department Biobehavioral Sciences and Psychology, Semel Institute for Neuroscience and Human Behavior, University of California, Los Angeles, Los Angeles, CA, USA; Department of Psychiatry, Brigham and Women’s Hospital and Harvard Medical School, Boston, MA, USA; Department of Psychology and Neuroscience, Temple University, Philadelphia, PA, USA; Department of Psychiatry, Yale University School of Medicine, New Haven, CT, USA; Connecticut Mental Health Center, New Haven, CT, USA; Department of Psychiatry, Perelman School of Medicine, University of Pennsylvania, Philadelphia, PA, USA; School of Psychology, Institute for Mental Health, University of Birmingham, Birmingham, UK; Centre for Human Brain Health, School of Psychology, University of Birmingham, Birmingham, UK; Centre for Youth Mental Health, The University of Melbourne, Parkville, VIC, Australia; Orygen, Parkville, VIC, Australia; Department of Psychiatry, McGill University, Montreal, Quebec, Canada; Program for Specialized Treatment Early in Psychosis (STEP), CMHC, New Haven, CT, USA; Department of Psychology and Neuroscience, Temple University, Philadelphia, PA, USA; Department of Psychology and Neuroscience, Temple University, Philadelphia, PA, USA; Department of Psychiatry, Washington University Medical School, St. Louis, MO, USA; Department Biobehavioral Sciences and Psychology, Semel Institute for Neuroscience and Human Behavior, University of California, Los Angeles, Los Angeles, CA, USA; Department of Radiology, Brigham and Women’s Hospital, Boston, MA, USA; Department Biobehavioral Sciences and Psychology, Semel Institute for Neuroscience and Human Behavior, University of California, Los Angeles, Los Angeles, CA, USA; Department of Psychology, Stanford University, Stanford, CA, USA; IBM T.J. Watson Research Center, Yorktown Heights, NY, USA; Department of Psychology, Northwestern University, Evanston, IL, USA; PEPP-Montreal, Douglas Research Centre, Montreal, Quebec, Canada; Department of Psychiatry, McLean Hospital and Harvard Medical School, Boston, MA, USA; Department of Psychiatry, Brigham and Women’s Hospital and Harvard Medical School, Boston, MA, USA; Department of Radiology, Brigham and Women’s Hospital, Boston, MA, USA; Department of Psychiatry, Icahn School of Medicine at Mount Sinai, New York, NY, USA; IBM T.J. Watson Research Center, Yorktown Heights, NY, USA; Centre for Human Brain Health, School of Psychology, University of Birmingham, Birmingham, UK; Department of Psychiatry, Semel Institute for Neuroscience and Human Behavior, University of California, Los Angeles, Los Angeles, CA, USA; Centre for Youth Mental Health, The University of Melbourne, Parkville, VIC, Australia; Orygen, Parkville, VIC, Australia; Department of Psychiatry, Brigham and Women’s Hospital and Harvard Medical School, Boston, MA, USA; Department of Psychiatry, Yale University School of Medicine, New Haven, CT, USA; Program for Specialized Treatment Early in Psychosis (STEP), CMHC, New Haven, CT, USA; Department of Psychiatry, Icahn School of Medicine at Mount Sinai, New York, NY, USA; Centre for Youth Mental Health, The University of Melbourne, Parkville, VIC, Australia; Orygen, Parkville, VIC, Australia; Department of Psychiatry, Perelman School of Medicine, University of Pennsylvania, Philadelphia, PA, USA; Department of Psychiatry, Yale University School of Medicine, New Haven, CT, USA; Connecticut Mental Health Center, New Haven, CT, USA; Orygen, Parkville, VIC, Australia; School of Psychology, University of New South Wales (UNSW), Kensington, NSW, Australia; Department of Psychiatry, Interdisciplinary Center Psychopathology and Emotion Regulation, University of Groningen, University Medical Center,Groningen, Netherlands; Department of Psychiatry, McLean Hospital and Harvard Medical School, Boston, MA, USA; Centre for Youth Mental Health, The University of Melbourne, Parkville, VIC, Australia; Orygen, Parkville, VIC, Australia; Orygen, Parkville, VIC, Australia; Division of Digital Psychiatry, Beth Israel Deaconess Medical Center, Harvard Medical School, Boston, MA, USA; John A. Paulson School of Engineering and Applied Sciences, Harvard University, Cambridge, MA, USA; Department of Psychiatry, McLean Hospital and Harvard Medical School, Boston, MA, USA; Department of Psychiatry, Center for Psychiatric Neuroscience, Lausanne University Hospital and University of Lausanne (CHUV-UNIL), Lausanne, Switzerland; Department of Psychological Medicine, Institute of Psychiatry, Psychology and Neuroscience King’s College London, London, UK; Centre for Youth Mental Health, The University of Melbourne, Parkville, VIC, Australia; Orygen, Parkville, VIC, Australia; Centre for Youth Mental Health, The University of Melbourne, Parkville, VIC, Australia; Orygen, Parkville, VIC, Australia; Renaissance Computing Institute, University of North Carolina, Chapel Hill, NC, USA; Department of Psychiatry, Beth Israel Deaconess Medical Center and Harvard Medical School, Boston, MA, USA; Department of Psychiatry, Beth Israel Deaconess Medical Center and Harvard Medical School, Boston, MA, USA; Department of Psychiatry, McLean Hospital and Harvard Medical School, Boston, MA, USA; Medical Informatics Fellowship, Veteran Affairs Boston Healthcare System, Boston, MA, USA; Food and Drug Administration, Silver Spring, MD, USA; Department of Psychiatry, Neurodevelopment & Psychosis Section, University of Pennsylvania, Philadelphia, PA, USA; Department of Psychiatry, Chonnam National University Medical School, Gwangju, Korea; Department of Psychology, Yale University, New Haven, CT, USA; Department of Psychology, Yale University, New Haven, CT, USA; Department of Psychiatry, IMHAY, University of Chile, Santiago, Chile; Centre for Youth Mental Health, The University of Melbourne, Parkville, VIC, Australia; Orygen, Parkville, VIC, Australia; Department of Psychiatry, Semel Institute for Neuroscience and Human Behavior, University of California, Los Angeles, Los Angeles, CA, USA; Department Biobehavioral Sciences and Psychology, Semel Institute for Neuroscience and Human Behavior, University of California, Los Angeles, Los Angeles, CA, USA; Department of Psychiatry, Yale University School of Medicine, New Haven, CT, USA; Connecticut Mental Health Center, New Haven, CT, USA; Department of Psychiatry, Donald and Barbara Zucker School of Medicine at Hofstra/Northwell, Hempstead, NY, USA; PEPP-Montreal, Douglas Research Centre, Montreal, Quebec, Canada; Department of Psychiatry, Chonnam National University Medical School, Gwangju, Korea; Mindlink, Gwangju Bukgu Mental Health Center, Gwangju, Korea; Department of Psychology, Northwestern University, Evanston, IL, USA; Department of Psychiatry, Neurodevelopment & Psychosis Section, University of Pennsylvania, Philadelphia, PA, USA; Centre for Youth Mental Health, The University of Melbourne, Parkville, VIC, Australia; Orygen, Parkville, VIC, Australia; Centre for Youth Mental Health, The University of Melbourne, Parkville, VIC, Australia; Orygen, Parkville, VIC, Australia; Department of Psychology, Emory University, Atlanta, GA, USA; Centre for Youth Mental Health, The University of Melbourne, Parkville, VIC, Australia; Orygen, Parkville, VIC, Australia; Department of Psychosis Studies, King’s College London, London, UK; Department of Brain and Behavioral Sciences, University of Pavia, Pavia, Italy; Department of Psychiatry, Brigham and Women’s Hospital and Harvard Medical School, Boston, MA, USA; Department of Brain and Behavioral Sciences, University of Pavia, Pavia, Italy; IRCCS Mondino Foundation, Pavia, Italy; Department of Radiology, Brigham and Women’s Hospital, Boston, MA, USA; Department of Psychiatry, IMHAY, University of Chile, Santiago, Chile; Department of Psychiatry, Brigham and Women’s Hospital and Harvard Medical School, Boston, MA, USA; Department of Psychiatry, IMHAY, University of Chile, Santiago, Chile; Department of Neurology and Psychiatry, Clínica Alemana—Universidad del Desarrollo, Santiago, Chile; Department of Psychiatry, Brigham and Women’s Hospital and Harvard Medical School, Boston, MA, USA; Centre for Youth Mental Health, The University of Melbourne, Parkville, VIC, Australia; Orygen, Parkville, VIC, Australia; Centre for Youth Mental Health, The University of Melbourne, Parkville, VIC, Australia; Orygen, Parkville, VIC, Australia; School of Psychology, Institute for Mental Health, University of Birmingham, Birmingham, UK; Centre for Youth Mental Health, The University of Melbourne, Parkville, VIC, Australia; Orygen, Parkville, VIC, Australia; Department of Psychiatry, IMHAY, University of Chile, Santiago, Chile; Department of Psychiatry, IMHAY, University of Chile, Santiago, Chile; Department of Brain and Behavioral Sciences, University of Pavia, Pavia, Italy; Department of Psychiatry, Semel Institute for Neuroscience and Human Behavior, University of California, Los Angeles, Los Angeles, CA, USA; Department Biobehavioral Sciences and Psychology, Semel Institute for Neuroscience and Human Behavior, University of California, Los Angeles, Los Angeles, CA, USA; Early Psychosis Detection and Clinical Intervention (EPIC) Lab, Department of Psychosis Studies, King’s College London, London, UK; Department of Psychiatry, Yale University School of Medicine, New Haven, CT, USA; Centre for Youth Mental Health, The University of Melbourne, Parkville, VIC, Australia; Orygen, Parkville, VIC, Australia; Department of Psychology and Neuroscience, Temple University, Philadelphia, PA, USA; Centre for Youth Mental Health, The University of Melbourne, Parkville, VIC, Australia; Orygen, Parkville, VIC, Australia; Centre for Youth Mental Health, The University of Melbourne, Parkville, VIC, Australia; Orygen, Parkville, VIC, Australia; Centre for Youth Mental Health, The University of Melbourne, Parkville, VIC, Australia; Orygen, Parkville, VIC, Australia; Department of Psychology, Stanford University, Stanford, CA, USA; Department of Psychiatry, Brigham and Women’s Hospital and Harvard Medical School, Boston, MA, USA; Department of Public Mental Health, Central Institute of Mental Health, Heidelberg Univeristy, Mannheim, Germany; Department of Psychiatry, Chonnam National University Medical School, Gwangju, Korea; Department of Psychiatry, Brigham and Women’s Hospital and Harvard Medical School, Boston, MA, USA; Early Psychosis Detection and Clinical Intervention (EPIC) Lab, Department of Psychosis Studies, King’s College London, London, UK; Centre for Youth Mental Health, The University of Melbourne, Parkville, VIC, Australia; Orygen, Parkville, VIC, Australia; Department of Psychiatry, Neurodevelopment & Psychosis Section, University of Pennsylvania, Philadelphia, PA, USA; Department of Psychiatry, Chonnam National University Medical School, Gwangju, Korea; Department of Psychiatry, Brigham and Women’s Hospital and Harvard Medical School, Boston, MA, USA; Department of Psychology and Neuroscience, Temple University, Philadelphia, PA, USA; Centre for Youth Mental Health, The University of Melbourne, Parkville, VIC, Australia; Orygen, Parkville, VIC, Australia; Department of Psychiatry, Brigham and Women’s Hospital and Harvard Medical School, Boston, MA, USA; School of Psychology, University of Birmingham, Edgbaston, UK; Centre for Human Brain Health, School of Psychology, University of Birmingham, Birmingham, UK; Centre for Youth Mental Health, The University of Melbourne, Parkville, VIC, Australia; Orygen, Parkville, VIC, Australia; Centre for Youth Mental Health, The University of Melbourne, Parkville, VIC, Australia; Orygen, Parkville, VIC, Australia; Department of Psychology, Stanford University, Stanford, CA, USA; Northwell Health, Glen Oaks, NY, USA; Department of Psychiatry, IMHAY, University of Chile, Santiago, Chile; School of Psychology, Institute for Mental Health, University of Birmingham, Birmingham, UK; Department of Psychiatry, IMHAY, University of Chile, Santiago, Chile; Department of Psychology and Neuroscience, Temple University, Philadelphia, PA, USA; Department of Psychiatry, Icahn School of Medicine at Mount Sinai, New York, NY, USA; Northwell Health, Glen Oaks, NY, USA; Department of Psychiatry, University of Pittsburgh School of Medicine, Pittsburgh, PA, USA; Centre for Youth Mental Health, The University of Melbourne, Parkville, VIC, Australia; Orygen, Parkville, VIC, Australia; Centre for Youth Mental Health, The University of Melbourne, Parkville, VIC, Australia; Orygen, Parkville, VIC, Australia; Department of Brain and Behavioral Sciences, University of Pavia, Pavia, Italy; IRCCS Mondino Foundation, Pavia, Italy; Department of Psychiatry, University of Hong Kong, Pok Fu Lam, Hong Kong; Department of Psychiatry, University of Hong Kong, Pok Fu Lam, Hong Kong; Department of Psychiatry, University of Hong Kong, Pok Fu Lam, Hong Kong; Centre for Youth Mental Health, The University of Melbourne, Parkville, VIC, Australia; Orygen, Parkville, VIC, Australia; Centre for Youth Mental Health, The University of Melbourne, Parkville, VIC, Australia; Orygen, Parkville, VIC, Australia; Centre for Youth Mental Health, The University of Melbourne, Parkville, VIC, Australia; Orygen, Parkville, VIC, Australia; Centre for Youth Mental Health, The University of Melbourne, Parkville, VIC, Australia; Orygen, Parkville, VIC, Australia; Department of Brain and Behavioral Sciences, University of Pavia, Pavia, Italy; Neuroradiology Department, IRCCS Mondino Foundation, Pavia, Italy; Department of Brain and Behavioral Sciences, University of Pavia, Pavia, Italy; Department of Psychiatry, University of Pittsburgh School of Medicine, Pittsburgh, PA, USA; Department of Brain and Behavioral Sciences, University of Pavia, Pavia, Italy; Department of Psychiatry, Brigham and Women’s Hospital and Harvard Medical School, Boston, MA, USA; Department of Psychology, Northwestern University, Evanston, IL, USA; Northwell Health, Glen Oaks, NY, USA; Centre for Youth Mental Health, The University of Melbourne, Parkville, VIC, Australia; Orygen, Parkville, VIC, Australia; Department of Psychiatry, IMHAY, University of Chile, Santiago, Chile; Department of Neurology and Psychiatry, Clínica Alemana—Universidad del Desarrollo, Santiago, Chile; Centre for Youth Mental Health, The University of Melbourne, Parkville, VIC, Australia; Orygen, Parkville, VIC, Australia; Department of Psychiatry, Neurodevelopment & Psychosis Section, University of Pennsylvania, Philadelphia, PA, USA; Department of Psychiatry, IMHAY, University of Chile, Santiago, Chile; Centre for Youth Mental Health, The University of Melbourne, Parkville, VIC, Australia; Orygen, Parkville, VIC, Australia; Centre for Youth Mental Health, The University of Melbourne, Parkville, VIC, Australia; Orygen, Parkville, VIC, Australia; Centre for Youth Mental Health, The University of Melbourne, Parkville, VIC, Australia; Orygen, Parkville, VIC, Australia; Department of Psychiatry, IMHAY, University of Chile, Santiago, Chile; Department of Psychiatry, IMHAY, University of Chile, Santiago, Chile; Department of Psychiatry, Brigham and Women’s Hospital and Harvard Medical School, Boston, MA, USA; Centre for Youth Mental Health, The University of Melbourne, Parkville, VIC, Australia; Orygen, Parkville, VIC, Australia; Department of Psychiatry, Brigham and Women’s Hospital and Harvard Medical School, Boston, MA, USA; Centre for Youth Mental Health, The University of Melbourne, Parkville, VIC, Australia; Orygen, Parkville, VIC, Australia; Unidad de imágenes cuantitativas avanzadas, departamento de imágenes, clínica alemana, universidad del Desarrollo, Santiago, Chile; Facultad de ciencias para el cuidado de la salud, Universidad San Sebastián, Campus Los Leones, Santiago, Chile; PEPP-Montreal, Douglas Research Centre, Montreal, Quebec, Canada; Department of Psychiatry, Semel Institute for Neuroscience and Human Behavior, University of California, Los Angeles, Los Angeles, CA, USA; Department Biobehavioral Sciences and Psychology, Semel Institute for Neuroscience and Human Behavior, University of California, Los Angeles, Los Angeles, CA, USA; Department of Psychiatry, Yale University School of Medicine, New Haven, CT, USA; Connecticut Mental Health Center, New Haven, CT, USA; Department of Psychiatry, Brigham and Women’s Hospital and Harvard Medical School, Boston, MA, USA; Department of Radiology, Brigham and Women’s Hospital, Boston, MA, USA; Department of Psychiatry, Massachusetts General Hospital and Harvard Medical School, Boston, MA, USA

**Keywords:** psychosis, clinical high risk, prediction, consortium, early detection, prevention

## Abstract

This article describes the rationale, aims, and methodology of the Accelerating Medicines Partnership® Schizophrenia (AMP® SCZ). This is the largest international collaboration to date that will develop algorithms to predict trajectories and outcomes of individuals at clinical high risk (CHR) for psychosis and to advance the development and use of novel pharmacological interventions for CHR individuals. We present a description of the participating research networks and the data processing analysis and coordination center, their processes for data harmonization across 43 sites from 13 participating countries (recruitment across North America, Australia, Europe, Asia, and South America), data flow and quality assessment processes, data analyses, and the transfer of data to the National Institute of Mental Health (NIMH) Data Archive (NDA) for use by the research community. In an expected sample of approximately 2000 CHR individuals and 640 matched healthy controls, AMP SCZ will collect clinical, environmental, and cognitive data along with multimodal biomarkers, including neuroimaging, electrophysiology, fluid biospecimens, speech and facial expression samples, novel measures derived from digital health technologies including smartphone-based daily surveys, and passive sensing as well as actigraphy. The study will investigate a range of clinical outcomes over a 2-year period, including transition to psychosis, remission or persistence of CHR status, attenuated positive symptoms, persistent negative symptoms, mood and anxiety symptoms, and psychosocial functioning. The global reach of AMP SCZ and its harmonized innovative methods promise to catalyze the development of new treatments to address critical unmet clinical and public health needs in CHR individuals.

## Introduction

The clinical high risk (CHR) for psychosis approach is used to prospectively identify people who are at increased clinical risk for developing psychotic disorders, ie, in the putatively prodromal phase of psychotic disorders.^1,2^ CHR criteria, which are based on a combination of attenuated symptoms, brief psychotic symptoms, and trait risk factors, are well-validated, reaching a good group-level prognostic accuracy (*AUC* = 0.85 at 34 months).^3^ Approximately one-quarter of CHR individuals convert to a psychotic disorder within a 3-year period, and 35% within 10 years.^4^ This rate is considerably higher than in the general population and other clinical populations, who have 0.5% and 3.9% likelihood of developing psychotic disorders over 3 years, respectively.^1,2,4–6^ The prevalence of CHR is 1.7% in the general population and 19.2% in clinical samples of youth.^7^ The CHR for psychosis criteria have been remarkably influential,^8^ with the Diagnostic and Statistical Manual of Mental Disorders-5 (DSM-5) including “Attenuated Psychosis Syndrome,” based on CHR criteria, as a condition for further study.^9,10^

Research to date, however, indicates substantial heterogeneity in clinical, neurocognitive, and neurobiological presentation, and trajectories and outcomes in the CHR population.^11^ Of CHR individuals who do not convert to psychosis, 48% experience full CHR remission at 12 months, and this number increases to 50% remission after 36 months.^12^ The remainder show progressing or persisting attenuated psychotic symptoms (APS),^13^ along with impaired functioning,^14^ cognition,^15^ and other symptoms.^16^ This high degree of heterogeneity in clinical course makes stratification of CHR individuals or prediction of individual-level outcomes using clinical information and/or biomarkers difficult but nonetheless of great importance. For example, it is challenging to match existing one-size-fits-all CHR interventions to the individual needs and phenotypes presented by CHR individuals and/or to develop new treatments that target individual pathoetiological mechanisms underlying variable CHR course. Reliable prognostic and predictive biomarkers are thus urgently required to meet these goals. In the current context, the term “biomarker” is used to refer broadly to cognitive, neuroimaging, digital health technology-derived, genetic, and biological markers.

Despite progress in identifying and validating predictive biomarkers across major international consortia,^17–19^ and good prognostic accuracy at the group level,^3^ current CHR prediction models do not reliably predict clinical course with the sufficient individual-level precision needed to yield clinically personalized approaches.^20^ Furthermore, the available individualized clinical prediction models are difficult to implement in real-world settings (fewer than 1% are being implemented in clinical care),^21^ largely because of heterogeneous assessment measurements. Thus, there is a need for well-powered, internationally coordinated efforts to better evaluate prospectively collected markers that can potentially inform interventional studies via state-of-the-art behavioral, neurobiological, and genetic measures. Such approaches would have 3 major benefits: (1) enhanced individual-level prediction of the full spectrum of outcomes in the CHR population; (2) harmonized assessment measurements that can facilitate transportability across global clinical settings and, therefore, support real-world implementation of precision psychiatry; and (3) identification and refinement of etiological mechanisms driving psychosis and psychiatric disorders more broadly. These elements are needed to develop novel and targeted precision therapeutics, to support the selection of primary clinical endpoints for future clinical trials, and to stratify patient samples optimally for clinical trials targeting those outcomes.

### Accelerating Medicines Partnership Schizophrenia

The Accelerating Medicines Partnership (AMP) is a public-private partnership between the National Institutes of Health (NIH), the U.S. Food and Drug Administration (FDA), the European Medicines Agency (EMA), and multiple public and private organizations. Managed through the Foundation for the NIH (FNIH), the AMP program aims to accelerate new and effect therapies to patients. AMP projects work toward this goal by identifying clinically relevant disease targets, improving identification of patients most likely to respond to particular treatment, and safely reducing the development timelines for life-saving therapies and improvements in patient outcomes.^22^

AMP Schizophrenia (AMP SCZ) marks the first AMP initiative directed towards a neuropsychiatric disorder. AMP SCZ partners include government (NIH, FDA, EMA), industry (Boehringer-Ingelheim, Janssen, Otsuka), and nonprofit (American Psychiatric Association Foundation, National Alliance on Mental Illness, One Mind, Schizophrenia, and Psychosis Action Alliance, Wellcome)^23^ partners. The approach is precompetitive, ie, research is conducted cooperatively without potential marketing activities or patenting, with the ultimate goal of accelerating the development of new therapeutics.

The goals of AMP SCZ are multifaceted, including: (1) developing measures that further define early stages of risk and prediction of the likelihood of progression to psychosis and other clinical endpoints; (2) generating tools that will facilitate the development of early-stage interventions to attenuate, delay, or prevent transition to psychosis in high-risk individuals; and (3) improving CHR clinical and functional outcomes. Such tools will be utilized in clinical trials to test new interventions for preventing or reversing various adverse outcomes in this population. AMP SCZ harnesses the power of open science to accelerate the research and development process and to advance promising therapies for CHR populations. As such, the planning and design of AMP SCZ addresses current recommendations for CHR prediction research, which include: (1) harmonizing CHR definition and assessment measures; (2) providing a data capture and aggregation infrastructure to support multiple data types and provide quality assessment; (3) harmonizing study design, measures, and infrastructure across research networks; (4) extending the scope of prediction beyond transition to psychosis; (5) creating collaborative, open-science research networks; and (6) creating common modeling platforms and open-source model libraries.^24^ The purpose of the current article is to provide a high-level overview of the aims and structure of AMP SCZ, with a summary of study design and methodology. Subsequent articles will be published providing further methodological detail about each data domain.

### Project Aims

The specific aims of AMP SCZ are to:

1. Establish a global research network

**Fig. 1.**
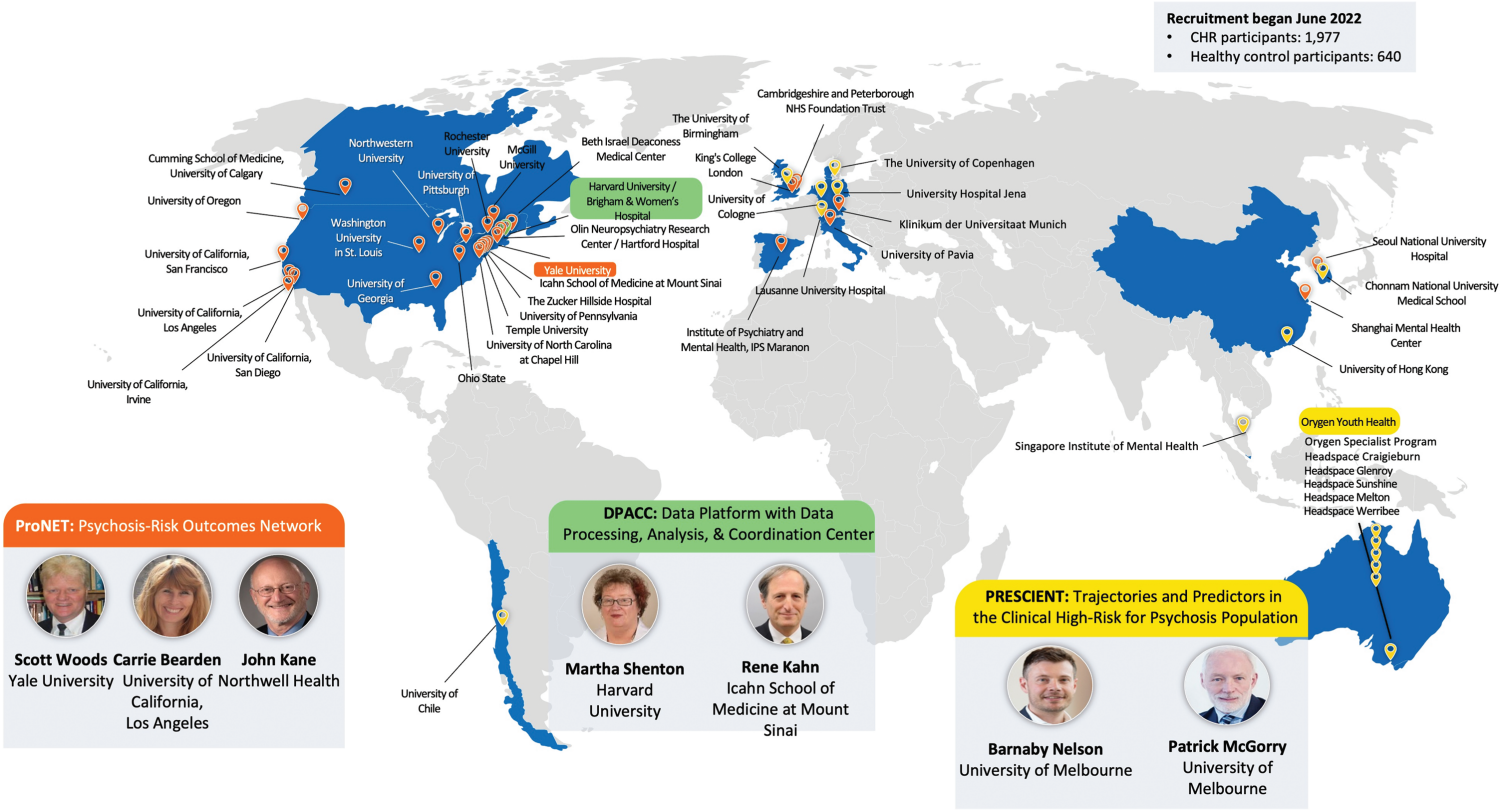
Accelerating Medicines Partnership Schizophrenia research networks and study sites.

AMP SCZ has established an international research network focused on recruiting young people at CHR for psychosis from 43 international study sites (see figure 1). The project’s large, international scope will ensure the generalizability of AMP SCZ findings.

2. Develop and validate tools for predicting individual outcomes

AMP SCZ will identify multimodal biomarkers that can be used to predict individualized clinical trajectories, including the likelihood of progression to psychosis, and illuminate heterogeneity within the CHR syndrome.

3. Set the stage for evaluating preventive as well as symptom and functioning-focused treatments

AMP SCZ will create a research framework that can improve clinical trial design and implementation, develop better approaches for measuring treatment response, and create more accurate tools for detecting early-stage risk. This will lay the foundation for the faster development of more effective treatments.

4. Create an accessible data repository

Data structures and dictionaries for each data type will be created to enable broad sharing. Data from AMP SCZ will be available to the scientific community through the National Institute of Mental Health (NIMH) Data Archive (NDA).

### Research Networks and Data Processing Analysis and Coordination Center

Two international networks have been established under the framework of AMP SCZ: Psychosis-Risk Outcomes Network (ProNET) and Prediction Scientific Global Consortium (PRESCIENT). Additionally, the Data Processing, Analysis, and Coordination Center (DPACC) will integrate and analyze the data generated in AMP SCZ and will utilize other key existing clinical high-risk cohorts to inform the AMP SCZ analyses. New tools developed for infrastructure pipelines and analyses will be made available to the scientific research community through the NDA. An overview of the AMP SCZ networks and study sites is provided in figure 1.

#### ProNET.

ProNET is led by S.W. Woods (Yale), C.E. Bearden (University of California, Los Angeles), and J.M. Kane (Zucker School of Medicine). This network comprises 28 international sites, including 19 in the United States, 2 in Canada, 3 in continental Europe, 2 in the United Kingdom, and 2 in Asia. ProNET is managed via a hub centered at Yale and Zucker.

#### PRESCIENT.

The PRESCIENT network is led by B. Nelson and P. McGorry (Orygen and University of Melbourne). The PRESCIENT network comprises 15 international sites, including the hub site in Melbourne, Australia (Orygen, consisting of the Orygen Specialist Program and 5 headspace clinics, *n* = 6), 3 in Asia, 4 in Europe, 1 in the United Kingdom, and 1 in South America.

#### DPACC.

DPACC is led by M.E. Shenton (Brigham & Women’s Hospital and Harvard Medical School) and R.S. Kahn (Icahn School of Medicine at Mount Sinai). The center is responsible for the data capture of information from ProNET and PRESCIENT networks, and setting up the infrastructure for data flow and data quality control, in addition to integration and analysis of the data, the latter in conjunction with members of the research networks and AMP SCZ partners. Members of DPACC are also leading analyses of key preexisting CHR-related datasets prior to applying them to AMP SCZ data. The DPACC team is divided into 4 Cores: (1) Coordination and Monitoring; (2) Data Management, Processing, and Archiving; (3) Data Analysis and Visualization; and (4) Dissemination of Resources, which includes developing and maintaining a website (ampscz.org) and making the tools developed available to the general research community.

### AMP SCZ Working Groups

Several working groups have been organized within AMP SCZ to oversee each assessment domain, including data capture and processing, clinical ascertainment and outcomes, electrophysiology, neuroimaging, cognition, genetics and fluid biomarkers, digital health technology-derived measures, and speech/facial expression (see table 1). Working groups for data analysis, co-enrollment, and outreach/dissemination of resources have also been established. The perspective of individuals with lived experience has been included throughout the AMP SCZ project, including the conception, protocol design, and ongoing conduct of the study.^25^ The co-chair of the AMP SCZ Steering Committee brings lived experience to the study, which is further supported by advocacy group members who serve on the AMP SCZ Steering Committee. Together, they supported the development of the AMP SCZ research plan and reviewed the study protocol yielding insight into assessment measures and perceived burden from the perspective of the study participant. Lived experience partners are involved in reviewing recruitment and the demographics of the sample to be consistent with the CHR population. Also, they present at AMP SCZ consortium meetings and ensure that the patient voice is incorporated in the project priorities to bridge the gap between science and real-world needs.^25^

**Table 1.** AMP SCZ Working Groups

Working Group	Leadership	Function
Team A: Data capture and processing pipelines	Sylvain Bouix	Develop overall architecture for data flow and management across research networks, DPACC, and NDA.
Team B: CHR ascertainment and clinical outcomes	Jean Addington Alison Yung	Develop ascertainment and clinical outcome measures. Monitor clinical training, ascertainment, and clinical assessments. Provide advice for data collection and preparation of data for the analysis of clinical measures.
Team C: Electrophysiology (EEG)	Daniel Mathalon Gregory Light	Develop and program EEG tasks, create training materials and Standard Operating Procedures (SOP) for set up and running of EEG sessions; create automated data processing pipelines that generate quality control measures, ERP waveforms, and topographic maps; develop web-based dashboard for display and review of each participant’s data to support QC and site-specific concerns, and to troubleshoot problems or challenges with EEG acquisition.
Team D: Neuroimaging	Michael Harms Ofer Pasternak	Develop MRI protocol for all MRI platforms, create training materials and SOP documents. Monitor ongoing data acquisition and work with sites to bring acquisitions into compliance following protocol deviations.
Team E: Cognition	Kelly Allott Bill Stone	Develop cognition assessment protocols; training and certification of assessors in cognitive assessments; monitoring and quality control; advise on outcome data and analyses.
Team F: Genetics and fluid biomarkers	Diana Perkins Scott Clark	Develop biosampling protocols and metadata; develop and monitor laboratory credentialing program; develop and review process quality benchmarks; provide technical advice for quality issues; develop genomic and fluid marker-related hypotheses and recommendations for biomarker selection.
Team G: Digital biomarkers	Justin Baker, John Torous	Develop and maintain digital phenotyping pipelines; train sites in mindLAMP (smartphone app) and use of a wearable device; ensure data quality, transform raw data into behavioral features.
Team H: Speech	Phillip Wolff, Guillermo Cecchi, Cheryl Corcoran	Create and automate a processing pipeline to extract linguistic, cognitive, emotional, and behavioral features from speech and facial data. Provide site training and certification for interviewing. Safeguard data quality at each step in the analysis.
Co-enrollment	John Kane	Review studies co-enrolling with AMP SCZ across recruitment sites to ensure AMP SCZ data integrity and minimize participant and rater burden.
Dissemination and Outreach	Tina Kapur, Eve Lewandowski	Develop and maintain processes and products to disseminate information from the AMP SCZ program to the broader community of researchers, clinicians, and families.

## Methods

### Establishing Harmonized Assessment Protocols Across Research Networks

The assessment domains, instruments, and measurement time points to be used across the AMP SCZ consortium were decided via Working Group discussions involving representatives from all stakeholder groups (members from the 2 research networks, DPACC, NIMH, and the AMP SCZ private and public partners). A consensus-based harmonized assessment battery was developed for use across sites in both research networks. This allows data to be pooled across all AMP SCZ sites, thereby increasing statistical power and generalizability of findings. The selection of assessment domains, measures, and time points was guided by the following principles: robust existing evidence regarding their relevance for CHR outcomes; capturing data that allows for both static and dynamic predictive modeling; timing of repeat assessments that would facilitate clinical trial design; balancing comprehensive data collection with participant and assessor burden; and innovative methods.

### Study Design

AMP SCZ is an observational longitudinal study examining clinical trajectories and predictors of clinical endpoints in the CHR population. Supplementary table S3 presents the schedule of assessments. The timing of outcome assessments was selected to span the breadth of clinical trial-relevant CHR endpoints. This includes attenuated positive, negative, and affective symptoms, general psychopathology, social and role functioning, CHR persistence/remission, transition to psychosis, and cognition over both the short (4 and 8 weeks) and longer term (12–104 weeks). A 2-month interval was chosen for a repeat of the biomarker assessments as there is evidence that change in biomarkers over this time period may be useful for outcome prediction.^26^ Additionally, change over this reasonably short time period would facilitate early stratification into subgroups for clinical trials.

### Sample

The AMP SCZ research network is recruiting a large cohort of CHR young people aged 12–30 years (*n* = 1977) and healthy control (HC) participants (*n* = 640) across 43 participating sites from 13 countries (figure 1). HC are matched to the sex, age, and parental socioeconomic status level of CHR participants enrolled at each site. HC participants complete screening, baseline, 12-month, and 24-month assessments, with a subset (5 of approximately 15 on average per site) completing additional key assessments at month 2 (see supplementary table S3).

### Inclusion and Exclusion Criteria

Inclusion and exclusion criteria are provided in table 2.

**Table 2.** Inclusion, Exclusion, and Conversion Criteria

*Inclusion Criteria* General inclusion criteria: a) Aged 12–30 years inclusive, b) Ability to give informed consent (parental/guardian consent is obtained for participants aged <18 years), and c) Meeting either CHR or healthy control criteria. CHR inclusion criteria: meet CAARMS-defined (Trait Vulnerability; Attenuated Psychotic Symptoms; Brief Limited Intermittent Psychotic Symptoms)^12^ or Structured Interview for Prodromal Syndromes^18^-defined (Brief Intermittent Psychotic Syndrome Current Progression; Attenuated Positive Symptom Syndrome Current Progression; Genetic Risk and Deterioration Current Progression) diagnostic criteria for CHR determined using a newly developed instrument, the *P*ositive *SY*mptoms and Diagnostic Criteria for the *C*AARMS *H*armonized with the *S*IPS (PSYCHS). Healthy control inclusion criteria: a) Do not meet CHR criteria or have a current or past Cluster A personality disorder, b) Not receiving any current treatment with psychotropic medication, and c) Do not have a family history (in first-degree relatives) of psychotic spectrum disorders.
*Exclusion Criteria* a) Antipsychotic medication exposure equivalent to a total lifetime haloperidol dose of >50 mg, estimated based on available information, or current antipsychotic medication at time of baseline assessment, b) Documented history of intellectual disability, c) Past or current clinically relevant central nervous system disorder, d) Traumatic brain injury rated 7 or above on the Traumatic Brain Injury screening instrument,^19^ or e) Current or past psychotic disorder.
*Conversion Criteria* a) At least 1 full threshold positive psychotic symptom as operationalized using the PSYCHS^18^ for 1 week or longer and occurring (i) for more than an hour a day, 3–6 days per week OR (ii) daily for less than 1 h, or b) At least 1 full threshold positive psychotic symptom with the above frequency but lasting less than 1 week in the context of newly prescribed or newly increased antipsychotic medication, or c) At least 1 full threshold positive symptom that is imminently dangerous (physically or to personal dignity or to social/family networks).

### Study Endpoints and Outcome Measures

The time periods of interest are 12 and 24 months post-baseline.

#### Primary Clinical Endpoint

##### Transition to Psychotic Disorder

As assessed by the *P*ositive *SY*mptoms and Diagnostic Criteria for the *C*AARMS *H*armonized with the *S*IPS (PSYCHS)^27^ by 12-month and 24-month follow-up, operationalized as per the definition in table 2.

#### Secondary Clinical Endpoints

##### Sustained Remission of CHR Status^11,13^

As defined by the PSYCHS^27^ for ≥6 months and until the last available follow-up.

##### Persistent CHR Status

Cases who do not meet sustained remission or transition criteria as assessed by the PSYCHS.

Other clinical endpoints of interest include relapse of CHR status, functional outcome, severity of attenuated psychotic symptoms, persistent functional impairment, persistent cognitive impairment, psychosocial functioning impairment, persistent negative symptoms, and incident and persistent nonpsychotic disorders.

### Measures

The full rationale and details of the measures across domains will be published elsewhere. A summary of key features is provided below.

#### Clinical Measures.

AMP SCZ represents the most unique, layered, and thorough clinical assessment to be included in a biomarker study, including multidimensional measures of clinical state at baseline, change over time, and clinical outcome. The key clinical measure is the newly developed PSYCHS,^27,28^ which is used to define CHR criteria, assess attenuated psychotic symptoms and determine the onset of a first-episode of psychosis. The PSYCHS was developed from the Comprehensive Assessment of At-Risk Mental States (CAARMS)^29^ and the Structured Interview for Psychosis-Risk Syndromes (SIPS)^30^ and will allow data from this project to be used in conjunction with legacy data that used these tools. The Structured Clinical Interview for DSM-5 (SCID-5)-Research Version (RV)^31^ is used to assess DSM-5 Mood and Substance Use disorders and the SCID-5 Personality Disorders^32^ to assess Schizotypal Personality disorder. If participants develop threshold psychosis, the Psychotic Disorder section of the SCID-5-RV is administered to determine the type of DSM psychotic disorder. Demographic information plus medical and psychiatric history, including prior and ongoing treatment, are also being collected.

A comprehensive assessment of psychopathology includes well-established measures such as the Brief Psychiatric Rating Scale,^33^ the Overall Anxiety Severity and Impairment Scale,^34^ the Columbia Suicide Severity Rating Scale,^35^ and the Alcohol, Smoking and Substance Involvement Screening Test.^36^ Of note are the Calgary Depression Scale for Schizophrenia^37^ to assess depression independently of negative symptoms, which has been validated for CHR,^38^ and the recently developed and tested Negative Symptom Inventory-Psychosis Risk (NSI-PR) to assess a range of negative symptoms.^39^

A focus on Patient Reported Outcomes (PROs) is rare in CHR longitudinal studies.^40^ However, several important PROs will be addressed: the Patient Global Impression of Severity to assess participants’ impression of the severity of their symptoms; the Patient Reported Outcomes Measurement Information System-Sleep Disturbance^41^ to measure sleep quality, depth, and restoration; the Perceived Stress Scale^42^; the Perceived Discrimination Scale^43^ to determine whether participants have experienced discrimination in their lifetime; the Pubertal Development Scale^44^ for determining the development of secondary sexual characteristics; and, lastly, the Psychosis Polyrisk Score^45^ to capture exposure to a range of environmental risk factors associated with psychosis.

Assessment of social and role functioning includes: The Social and Occupational Functioning Assessment Scale,^46^ the Premorbid Adjustment Scale,^47^ and the Global Functioning (GF): Social and Role Scales.^48^ The GF scales each provide a single summary score that accounts for age, reflects change over time, and avoids confounding with psychiatric symptoms.

#### Digital Health Technologies-Derived Measures.

Digital health technologies (DHT)-derived measures are an optional aspect of the study. Informed by feedback from young people with lived experience of CHR and co-designed with their input, the study offers the following DHT-derived measures:

[1] Daily surveys: a self-report questionnaire consisting of 30 short questions on thoughts, feelings, and behaviors of the past day (eg, “Today I felt down”) is assessed every evening for 1 year on participants’ smartphone via the open-source mindLAMP app. The daily questions capture multiple domains (eg, mood, psychotic experiences, social interactions, and events). Participants can also record up to 2 min of spoken voice diaries. The surveys are available in 9 languages and the app captures relevant metadata such as response latency. Survey responses are plotted and shared in real-time with participants via the app. All data are automatically and securely transferred to study servers to enable remote research.
[2] Passive sensing data: Participants can choose to share passive sensing data gathered from their smartphone through the same mindLAMP app, namely geolocation (GPS), accelerometer, and/or screen-state (on/off) data. Participants can choose to share all data listed above, opt out of GPS, or use the app only for daily surveys. mindLAMP data are uniquely supported by a flexible Application Programming Interface (API). Digital phenotyping data can also be shared back when processed into behavioral and clinical features including metrics related to hometime, steps, sleep duration, sedentary periods, duration of screen use, and environmental exposure (eg, green space, population, etc.).^49^
[3] Actigraphy: Participants wear Axivity AX3 (Newcastle upon Tyne, UK) watches (accelerometers assessing 24h gross motor activity) during the first year to measure rest-activity patterns and physical/sedentary behaviors. Data are transferred to institutional servers at study visits.

#### Cognition.

The Wide Range Achievement Test 5 Reading subtest^50^ is used to estimate premorbid IQ at English language sites. However, as some countries do not typically measure reading accuracy and given the task will vary based on language, some sites are using a local version of a reading task, and others do not measure premorbid IQ. In English-speaking countries, the 2-subtest version (Vocabulary and Matrix Reasoning subtests) of the Wechsler Abbreviated Scale of Intelligence-Second Edition^51^ (WASI-II) is used to measure current Full-Scale IQ. As the WASI-II is only available in English, non-English-speaking sites are administering, in their local language, the Vocabulary and Matrix Reasoning subtests of the Wechsler Adult Intelligence Scale–Fourth Edition^52^ (WAIS-IV) for participants aged ≥16 and the Wechsler Intelligence Scale for Children–Fifth Edition^53^ (WISC-V) for participants aged <16 years.

Using the Penn Computerized Neurocognitive Battery^54^ we are measuring the following specific cognitive domains: attention (Short Penn Continuous Performance Test), working memory (Short Fractal N-Back Test), processing speed (Digit-Symbol Test), relational memory (Digit-Symbol Recall), verbal learning (Short Penn List Learning Test), visual memory (Short Visual Object Learning Test), emotion recognition (Penn Emotion Recognition Test), motor function (Short Computerized Finger-Tapping Test), and sensorimotor speed (Motor Praxis Test).

#### Electrophysiology.

To minimize cross-site differences in EEG hardware, identical high-impedance active electrode EEG recording systems (BrainProducts actiChamp 64-Channel system^55^) were leased from a vendor (Neurosig, Inc.) that also: (1) custom engineered a stimulus delivery system dedicated to the AMP SCZ paradigms that presented auditory and visual stimuli with high temporal precision, and (2) developed a software interface for EEG acquisition laptops that included set-up instructions for EEG technicians and participant task instructions in the local language. EEG measures were selected based on their previously established sensitivity to schizophrenia and/or ability to predict CHR clinical endpoints. They include: (1) Auditory mismatch negativity to pitch + duration “double-deviant” tones^56,57^ recorded concurrently while the participant performs a primary visual oddball task, thereby allowing simultaneous assessment of (2) Visual P300 event-related potentials (ERPs)^56,58^ to infrequent target circles (P3b) and novel fractal images (P3a); (3) Auditory P300 ERPs^58,59^ to infrequent target tones (P3b) and novel sounds (P3a); (4) Gamma oscillations assessed with 40-Hz auditory steady-state responses^60,61^; and (5) 1/f periodic and aperiodic components of EEG power spectra^62^ assessed from resting EEG (eyes open/closed). See supplementary materials for further details.

#### Neuroimaging.

The multimodal neuroimaging protocol was designed for a 3T MR scanner and includes structural imaging, resting-state functional MRI (rfMRI), and multi-shell diffusion-weighted scans. There are currently 37 MR scanning sites with a variety of vendors and platforms: Siemens Prisma (*n* = 28), Siemens Skyra (*n* = 4), Siemens Vida (*n* = 1), General Electric MR750 (*n* = 3), and Philips Achieva DDAS (*n* = 1). All sites use either 32 or 64-channel head coils. The protocol represents a synthesis of aspects of the HCP-Lifespan and ABCD Study protocols,^63,64^ with a cumulative scan duration of approximately 50 min. Specifically, it includes 0.8 mm isotropic structural scans (3D T1-weighted and T2-weighted scans, each ~6–7 min); 2.4 mm isotropic resting-state BOLD (acquired as 4 × 5 min runs with *TR* = 900 ms, *TE* = 35 ms, and multiband-factor = 6), and a 1.8 mm isotropic multi-shell diffusion scan (9–10 min, multiband-factor = 3). The structural and rfMRI scans are relatively well harmonized in terms of basic scan parameters across vendors and platforms, which was made possible without sacrificing much performance on the Prisma scanners, since those modalities are not dramatically affected by peak gradient strength.

However, since the different 3T MR platforms in AMP SCZ have considerable differences in peak gradient strength (Siemens Prisma: 80 mT/m; Siemens Skyra: 45 mT/m; Siemens Vida: 60 mT/m; General Electric MR750: 50 mT/m; Philips Achieva DDAS: 40 mT/m), which is particularly important for diffusion imaging, we created a “two-tier” protocol to take full advantage of the Prisma’s higher peak gradient strength. Specifically, on the Prisma, we are collecting shells with b-values of 200, 500, 1000, 2000, and 3000 s/mm^2^ (6, 10, 50, 50, 50 directions, respectively; *TR* = 3200 ms; *TE* = 79.4 ms). On all the other platforms we are collecting the same diffusion directions, but only for the *b* = 200, 500, 1000, and 2000 s/mm^2^ shells. Thus the non-Prisma diffusion data represents an exact *subset* of the Prisma diffusion protocol (albeit acquired with a longer *TR* and *TE* as well; *TR* = 3970–4250 ms; *TE* = 96–97 ms). This provides several flexible options for how the diffusion data can be analyzed. To promote consistent acquisition of the protocol, the Siemens protocol is highly automated with field-of-view positioning and orientation handled automatically using Siemens “AutoAlign” feature on all platforms (Prisma, Skyra, and Vida). On the other platforms, positioning, and orientation are set manually by the scan operator and linked (copied) to later scans in the session where appropriate. See supplementary materials for details of neuroimaging data flow and quality assurance procedures.

#### Genetics and Fluid Biomarkers.

Blood and saliva samples are collected at baseline and at 2-month follow-up. Blood is processed using a standardized protocol to obtain aliquots of platelet-poor plasma, serum, and whole blood. In addition, the buffy coat is aliquoted for DNA extraction. Samples are stored in a minus 80 freezer within 90 min of collection. In addition, 3 saliva samples are collected over a 2h period. Relevant metadata includes height, weight, sleep and awakening times, time fasting, recent illnesses or inflammatory conditions (eg, fever, respiratory systems, acne, sunburn, white blood cell differential), and physical activity.

We intend to analyze potential biomarkers of psychosis risk, especially those biomarkers that might reflect 1 or more treatment targets or be useful in risk stratification. Salivary cortisol^65–69^ will be assessed and DNA will be analyzed to obtain polygenic risk scores (PRS) for schizophrenia and other conditions.^70,71^ As study participants are intended to be ethnically diverse we propose to generate PRS with the Blended Genome Exome (BGE) assay, recently developed at the Broad Institute. The BGE is a state-of-the-art, cost-effective approach to capturing genetic diversity that blends high-pass sequencing of protein-coding and low-pass sequencing of non-protein-coding regions. Additional promising biomarker candidates under consideration include those reflecting redox system function,^63^ immune system dysregulation,^64,65^ and fatty acid levels.^70^ While each of these groups of markers has been associated with risk of transition to psychosis, there is emerging evidence for interaction between genotype, aberrant immune response, redox status, and membrane fatty acid composition, with subsequent impact on brain connectivity and plasticity and on dopamine and glutamate systems related to the underlying pathology of psychotic illness.^72,73^

All remaining fluid and DNA biospecimens will be stored indefinitely at the NIMH Repository and Genomics Resource (NRGR) for future research. See supplementary materials for further details.

#### Spoken Language and Facial Expression Samples.

Spoken language and facial expression samples are obtained from open-ended conversations (~20 min) and semi-structured PSYCHS interviews using Zoom’s online meeting platform. Spoken language samples are also acquired from audio diaries captured over smartphones. Face processing uses the open-source software packages Py-Feat^74^ v0.5 and MediaPipe v0.9.3.0^75^ to determine the number and location of faces in a video. The landmark detection algorithms in these packages support the extraction of facial action units,^76^ allowing for the detection of common expressions and emotions. Zoom is parameterized to create separate audio files for each speaker in an online meeting. The open-source software packages Praat v6.3.10^77^ and openSMILE v3.0.^78^ are used to extract acoustic features such as voice stability, noise measurements, pitch variations, spectral characterizations, vowel space, and timbre features.

The 3 kinds of spoken language samples are converted into text using the TranscribeMe!^79^ transcription service. The transcripts are full verbatim: they include everything that is said, including filler words, false starts, grammatical errors, and nonlinguistic expressions, such as sighs and laughs. The service’s HIPAA-compliant workflow ensures proper handling of sensitive medical information. Human-editors identify personal information (personal health information and personally identifying information) so that it can be redacted from the transcript. Sentence-level timestamping enables time-course analyses of linguistic features over the course of an interview. The same transcription service is used for all 8 language communities included in the study.

Language recordings reflecting people’s naturally occurring thoughts, temporally aligned with facial expressions, acoustics, and prosody, will enable recovery of the concepts behind the words, the semantics of the face, and the hidden meaning of speech sounds.

#### Data Flow and Quality Assessment.

**Fig. 2.**
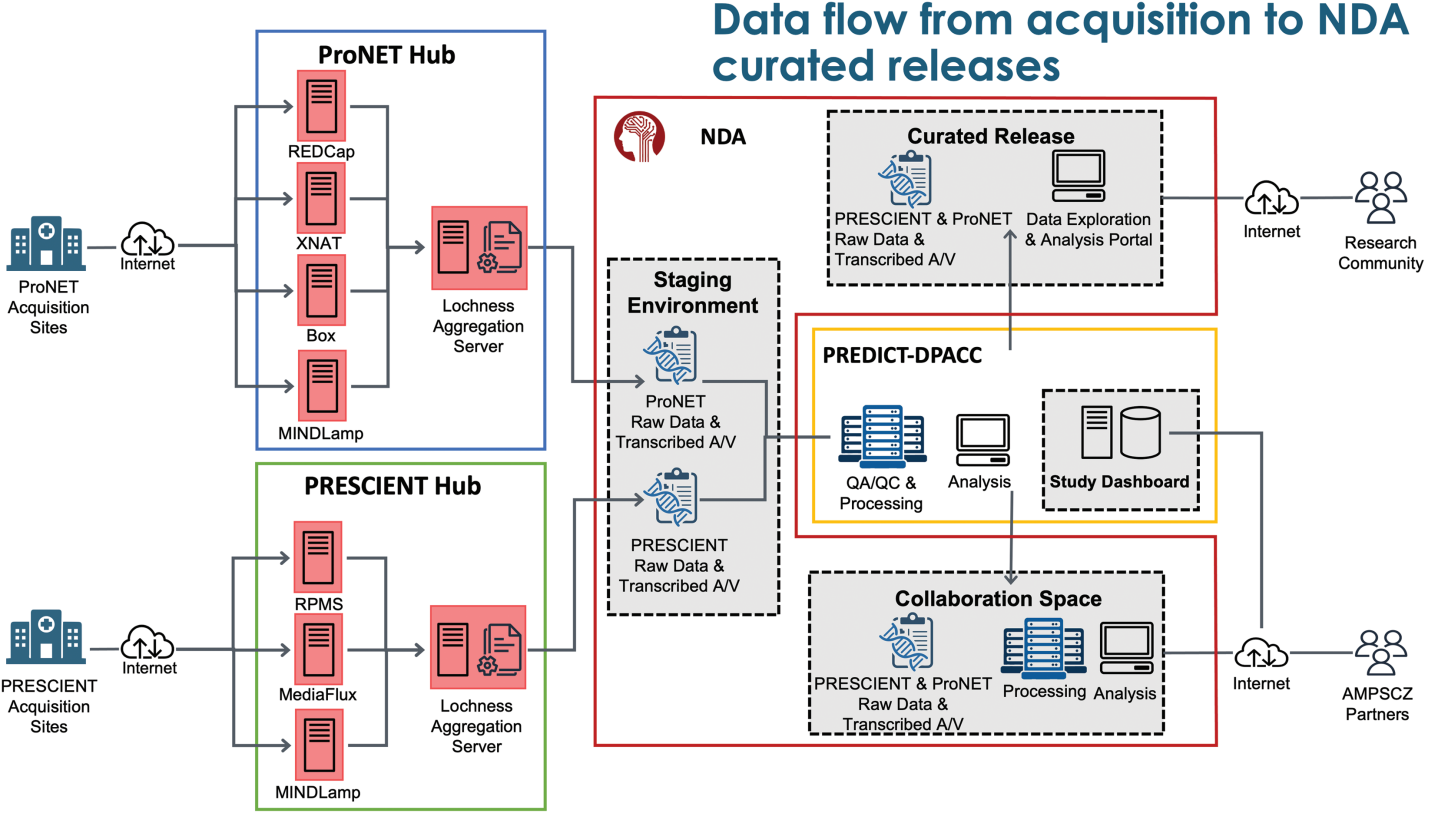
Overview of the data flow from acquisition to National Institute of Mental Health (NIMH) Data Archive (NDA) curated releases.

Figure 2 shows data flow from acquisition to the NDA curated releases. The data flow for this project is unique in that it requires coordination between 4 entities: 2 research networks each managing separate data capture systems, the DPACC overseeing data processing, QC pipelines and data visualization, and the NDA where curated data is deposited and disseminated. ProNET manages 4 data capture systems: REDCap for form data, XNAT for MRI data, MINDLamp for phone data, and Box for other file transfers (video recordings, actigraphy data, EEG scans). PRESCIENT manages 3 systems: RPMS for form data, MINDLamp, and MediaFlux for all other data types. Both networks use the PennCNB system to administer neurocognitive tests to participants. Each network also maintains a data aggregation and de-identification server, which pulls data from their data capture systems, organizes the data files on a local filesystem, and then performs de-identification procedures. For the AMP SCZ project, de-identification primarily consists of extracting key features from audio/video recordings, including redacted transcripts as raw data cannot leave the networks. These include automated QC as well as visual inspection of all data types. Key QC metrics and study monitoring information are displayed in a custom study dashboard hosted by the DPACC.

Aggregate and de-identified data are automatically transferred twice-daily to a staging area at the NDA, from which the DPACC then pulls the data for review, validation, quality checking, and further processing. Data are then further transformed to meet the standards established by the NDA data dictionaries and uploaded to the NDA collaboration space,^13^ where data can be explored and analyzed by AMP SCZ partners in Amazon workspaces virtual desktops. Data will also be shared with the wider community via traditional NDA data releases. Network-specific data flow and data quality checking is described briefly in supplementary materials.

Data quality is assessed in near real-time for all data types. Form data such as clinical interviews or cognitive tests are checked for data missingness and valid ranges. Automated and manual QC pipelines have been established for all complex data types including EEG, MRI, A/V, fluid, and digital biomarker data, monitored by DPACC and the relevant data type experts from the wider AMP SCZ community. Run sheets are checked for any protocol deviation or data quality issues observed by study staff during data acquisition. All QA/QC findings are reported back to the research networks management team for follow-up with the individual data collection sites. By assessing data quality in near real-time, missing or poor quality measures can often be corrected or re-collected. Additionally, deviations in protocols are caught quickly and can be amended. Finally, if the wrong protocol is run, or corrupted, because it is in near-time, the site can be contacted to rerun the participant for that measure.

#### Sample Size and Power.

The CHR sample size is primarily determined by the aim of developing a prediction model for transition to psychosis by 12 months and 24 months. We have used a recently described method^80^ for developing a clinical prediction model using a traditional likelihood-based approach (eg, logistic regression) to estimate an appropriate sample size. Specifically, sample sizes were derived for the scenarios of having low, medium, or high prediction performance and also for the number of parameters in the model being 10, 20, 30, 40, or 50. The expected transition rate used was 15% over 1 year. Assuming a transition rate of 15%, and a maximum of 30 parameters included in the predictive model, the minimum sample size for a model is estimated to be between 1100 and 1300 for a survival outcome (ie, an outcome that accounts for time to conversion and censoring; see supplementary material). Our projected sample size of 1977 CHR young people is therefore adequate for developing a prediction model with ~30 parameters that has high predictive performance, while still allowing a subset of the data to be “held back” for internal validation purposes. The adequacy of this sample size for machine-learning-based prediction models was confirmed in the simulation analyses of PRONIA data.^81^ The size of the HC sample was determined primarily to control for site effects, particularly for diffusion MRI data which is highly sensitive to scanner biases.^82,83^ Simulation analyses in the PRONIA^18^ dataset suggest that 15 HC per MRI scanner is recommended to control for site effects in diffusion data.

#### Data Analysis.

Data analysis for AMP SCZ will be performed by the Analysis and Visualization core of the DPACC in consultation with the Data Analytic Strategy Workgroup, which includes members from all consortium parties. The analyses will focus on 2 main goals: (1) prediction of clinical endpoints^18,81,84–88^ and (2) characterization and prediction of clinical trajectories.^89,90^ These goals will be achieved using state-of-the-art machine-learning methods that are robust, flexible, and clinically informed.^91,92^ Predictions will be based on data collected at baseline until and including the month 2 follow-up visit. This approach was chosen to optimize the usability for potential dynamic participant selection procedures in future clinical trials, while leveraging the expected stronger prediction value encapsulated in the longitudinal data.^93–95^ To ensure our ability to test the robustness of prediction models in an unbiased manner, 200 CHR individuals (roughly 10% of the entire data) will be held out from distribution for use as an unseen independent validation sample. This size of a held-out set has the power to validate predictors with area under the curve of 0.7 or higher, and with a prevalence of 10% or higher.^96,97^ Flexibility will be assured by applying data-fusion techniques^81,98–101^ to support the multimodal and longitudinal data. Our approaches will be clinically informed by implementing Explainable artificial intelligence (AI) methods^102^ that derive informative predictions that can be mapped back to individual features and biomarkers, as opposed to some deep learning and other “black-box” techniques that may not be appropriate for achieving interpretability. Clinical trajectory analyses, utilizing data from baseline, 12- and 24-month follow-up, will identify biomarkers and outcome measures that are associated with the endpoints so that they can inform future clinical trials, help construct risk calculators, and provide a more mechanistic understanding of the clinical endpoints using Explainable AI techniques.^102^ Trajectory analyses will include all information available from all time points, and will apply state-of-the-art clustering and latent variable analyses^103–106^ designed to find subtypes of CHR, and to identify measures that are informative regarding the endpoints, and that may be useful as biomarkers that will further facilitate population enrichment in future clinical trials. These analyses also lay the foundation for developing individual treatment paths, where prediction models in future clinical trials can be trained to identify individuals more likely to benefit from specific treatments, and trajectory models can facilitate dynamic models that could inform clinical decisions at each evaluation point.

#### Dissemination of Resources.

A website has been created (ampscz.org) that provides an overview of the aims and activities of the program for use by researchers, potential help-seeking youth and their caregivers, and clinicians looking for clinical trials that may be of help for their patients. The details for each study site in the networks are provided in the website, including contact information for all the principal and co-investigators, as well as the tools, protocols, standard operating procedures, and workflows. Tutorials will also be added to the website as they become available. The source code for the tools we have been building is available on GitHub (https://github.com/AMP-SCZ/). An annual meeting brings together AMP SCZ stakeholders across the networks and the DPACC, and includes presentations by senior scientists, junior researchers, public, and private partners, and experts with lived experience to disseminate information about progress toward project goals and other information relevant to stakeholders. Participation of AMP SCZ teams in an established international alliance of open-source software engineers and scientists will be organized to share best practices in the harmonization and analysis of multimodal data collected from multi-institutional international studies.

## Discussion

With the goal of recruiting approximately 2000 CHR young people, AMP SCZ will be the largest and most diverse cohort study of individuals at CHR for psychosis to date, ensuring appropriate power and generalizability of findings. Identifying antecedents of psychosis and other outcomes has important implications for early detection, prevention, and treatment of psychotic disorders. Given its large projected sample size and geographical reach, AMP SCZ will develop and validate effective methods for dissecting the heterogeneity of CHR trajectories. This is critically needed in order to support the selection of primary outcome measures for future clinical trials, to stratify patients according to risk level for outcomes of interest, and to provide insight into pathoetiological mechanisms that can be targeted in future studies of novel treatments. This will accelerate targeted early intervention strategies, based on clinically relevant predictive models, which are critical to prevent psychosis onset and other adverse outcomes in CHR patients.

AMP SCZ will establish a landmark dataset for current and future analyses related to psychosis risk. The design of the project was guided by a number of innovative approaches, including: (1) a large and diverse international CHR sample recruited across 16 countries, ascertained using a newly developed instrument (PSYCHS) that harmonizes the 2 most widely used instruments in the field (ie, CAARMS and SIPS); (2) inclusion of the perspective of individuals with lived experience in the protocol design phase and in the ongoing conduct of the study; (3) a repeatable core set of clinical outcome measures suitable for future clinical trials and empirical trajectory analyses; (4) real-time behavioral data from smartphone sensors and actigraphy; (5) symptom reports from surveys that offer novel longitudinal and dynamic data on clinical, functional, and cognitive outcomes that will provide a rich new data set to deconstruct clinical heterogeneity; (6) a novel neuropsychological assessment battery designed to combine computerized and paper-and-pencil measures in a manner that provides sensitive, remote, longitudinal, international assessment of at-risk cognitive domains across multiple languages and cultures, while also minimizing participant burden; (7) a panel of multimodal measures collected at 2 early timepoints suitable for use in patient stratification based on dynamic prediction of subsequent outcomes and trajectories; (8) modern “HCP-Style” MRI acquisition protocol harmonized across scanner platform for assessment of both structural and connectivity changes, with certification of sites in implementation of the protocol; (9) leasing of identical EEG acquisition systems and custom-engineered stimulus delivery systems dedicated exclusively to the AMP SCZ project, as well as implementation of an automated EEG processing pipeline and web-based dashboard displaying single subject data and quality control metrics that are updated daily and reviewed in weekly video calls with the EEG technicians and investigators from AMP SCZ sites; (10) partnership with the Broad Institute using state-of-the-art genotyping that is designed to better account for genetic diversity, addressing a critical issue in polygenic risk determination; (11) language samples, in sync with facial expressions and speech characteristics, are collected and analyzed using Artificial Intelligence, enabling the extraction of underlying concepts, emotional cues, and semantic information from both verbal and non-verbal communication; (12) close to real-time quality control of all collected data and reporting of issues back to sites so that errors can be quickly corrected and incorporated into future data collection, and in some instances an error in a measure can be rerun so that correct data replace the incorrect data; (13) incorporation of a novel poly-environmental risk score into multimodal analyses; and (14) a cutting-edge data capture and study management system that is flexible, scalable and customizable, and can support multiple data types, including traditional types such as questionnaires and imaging data, but also emerging data types from phones, personal tracking devices, and audio-visual recordings.

The global reach of AMP SCZ and its harmonized innovative methods promise to catalyze efforts to address critical unmet clinical and public health needs in CHR patients. This initiative will provide tools that will enable testing of more precise and mechanism-linked treatments and advance the goal of averting the onset of psychotic disorders in high-risk individuals.

## Supplementary Material

sbae011_suppl_Supplementary_Material
