# Supplementary material for: Accelerating Medicines Partnership® Schizophrenia (AMP® SCZ): Rationale and Study Design of the Largest Global Prospective Cohort Study of Clinical High Risk for Psychosis
Source: Schizophr Bull. 2024 Mar 7;50(3):496–512. doi: 10.1093/schbul/sbae011 (PMC11059785; doi:10.1093/schbul/sbae011)
Supplement: sbae011_suppl_Supplementary_Material [file sbae011_suppl_supplementary_material.docx]

**Accelerating Medicines Partnership® Schizophrenia (AMP® SCZ):**

**Rationale and Study Design of the Largest**

**Global Prospective Cohort Study of Clinical High Risk for Psychosis -**

**Supplementary Material**

**Methods**

Clinical measures

*Cross-site reliability of clinical assessments*

A rigorous process for ensuring cross-site reliability is being followed. Research assistants across each site complete an extensive training program and need to be certified on each of the clinical measures before collecting data. Training materials were developed by our clinical ascertainment and outcomes working group (Team B/I). For each measure, trainees score a mock participant and their scores are compared against ‘gold standard’ scores for that measure. Trainee raters need to meet a reliability threshold (≥0.80) and typically need to have rated the mock participant within one point of the gold standard. Trainees who do not meet these criteria are asked to re-rate or rate a new mock participant (depending on the measure). For the SCID, research assistants are also required to administer an interview with a mock participant. A certifier sits in on that mock interview and rates the trainee’s competence in administering the SCID.

All of the clinical measures for which there was not already a translated version available were translated and back-translated by an official translation service to ensure accuracy. Where possible (e.g., the SCID) we used the official copyright version of the measure in each language.

In addition to these training and translation procedures, there are multiple study processes that ensure the reliability of clinical scale ratings across sites:

- Regular ‘consensus calls’ are scheduled for the purpose of discussing scores on the PSYCHS, a key clinical measure in AMP SCZ. These consensus calls are led by leaders in the field. Their purpose is to discuss and reach consensus on PSYCHS ratings from screener assessments, in order to confirm eligibility/ineligibility for the study, and on follow up assessments where the participant may have met conversion criteria, in order to reach consensus on determination of conversion status. Manuals for each clinical instrument have been developed and made available (e.g., PSYCHS manual, NSI-PR manual, SCID manual).
- Administration and scoring of measures are discussed as needed in monthly network meetings.
- Research assistants have weekly 'scoring' sessions where they can discuss any scoring questions/difficulties they are having with the team.
- In fortnightly team meetings, there is a standing item on the agenda for scoring questions where research assistants can add their scoring questions to discuss with the broader team, or where project managers can address questions that they have been frequently asked by research assistant staff.

Detailed information about these procedures will be included in the forthcoming methods paper focused on clinical assessments in AMP SCZ.

*Substance use*

There is a high prevalence of substance use/dependence in the CHR population.[^1^](https://paperpile.com/c/zes0fh/0mhCj) Therefore, excluding on this basis would result in an unrepresentative sample, limiting the generalizability of AMP SCZ findings. We have therefore not included current substance dependence as an exclusion criterion. However, we note that in order to meet CHR criteria using the PSYCHS instrument, attenuated psychotic symptoms need not to be better explained by another DSM-5 disorder (such as substance dependence) or to occur only during peak intoxication from hallucinogens, amphetamines or cocaine. The former corresponds to SIPS CHR criteria and the latter to CAARMS CHR criteria. Furthermore, it is possible that there will be region-dependent differences in substance use across study sites. In order to ensure that these differences are well characterized, we are collecting detailed information about substance use from all participants using the ASSIST instrument and the DSM-V substance use module. This will allow researchers to interrogate the variability of substance use/dependence across sites and the possible impact of this on other variables and outcomes of interest.

Digital measures

MindLAMP supports multimodal digital phenotyping data. While not all features of mindLAMP are utilized in AMP SZ, understanding the full potential of the app is important to understand the broader potential of this data and its clinical relevance.

*1-Data Capture Capabilities*

mindLAMP is uniquely able to capture diverse data streams that enable it to function concomitantly as a simple EMA app and a robust digital phenotyping tool. Types of data collection are noted below:

- Passive data derived from smartphone sensors including accelerometer, gyroscope, screen-time, geolocation, call/text logs, phone charging time, and more are all available for data collection with mindLAMP.[^2^](https://paperpile.com/c/zes0fh/IUEdl) mindLAMP is also uniquely able to capture Apple HealthKit, Apple SensorKit, and Google Fit data directly from smartphones given our team’s adherence to these emerging standards. Data processing pipelines enable transforming this raw data into clinically meaningful constructs through custom pipelines, including, such as geolocation data into features relevant to social functioning and social rhythms.[^3^](https://paperpile.com/c/zes0fh/ARrT5)
- Wearable data from common devices like the Apple Watch or Wear OS by Google Smartwatch is supported by mindLAMP. While AMP SZ uses a wearable which does not support either Apple or Google APIs, the ability to use diverse devices with mindLAMP offers potential for future related research.
- EMA and survey data can be scheduled or available on demand. Unique features of EMA and survey data on mindLAMP include the ability to capture metadata such as response latency with utility highlighted in a paper on assessment of suicidality examining response to question-9 on the PHQ-9 depression screener.[^4^](https://paperpile.com/c/zes0fh/56L7d)
- Cognitive tests related to attention and memory – such as Trails A/B, and spatial span – are already available in mindLAMP with early research showing utility of these assessments.[^5^](https://paperpile.com/c/zes0fh/3xDKp) While these are not utilized in AMP SZ, others can employ them today in research.

*2-Flexible and Customizable Backend*

The flexible and customizable backend of mindLAMP offers important benefits. Each site can easily run the common study protocol in terms of data capture, but also to add new site-specific questions or schedules if desired. The backend of mindLAMP allows each site to store its own data centrally or locally, meaning that the app can be run in a distributed manner as needed to comply with local, state, federal, and international laws. As necessary, mindLAMP can also be deployed from a central site to support teams not wishing to locally administer it.

*3-Open Source and Sharable Code Base*

The open-source nature of mindLAMP ensures that anyone across the world can work with, expand, and use the platform. The code is available on GitHub (<https://github.com/BIDMCDigitalPsychiatry>). This represents a healthy software ecosystem that ensures findings from research on mindLAMP are real-life tools that others will engage with and develop further.

*4-Alignment with Apple and Google Best Practices*

mindLAMP is uniquely aligned with both Apple and Google regulations and priorities around app use for clinical research and care. It does not violate Apple or Google regulations around use of data (eg apps that report on screen activity) and thus does not risk immediate removal from the app stores. mindLAMP has received special permission from Apple to support Apple SensorKit.[^6^](https://paperpile.com/c/zes0fh/Ft7mr) While SensorKit is not utilized in AMP SZ, it offers important future proofing protection.

*5-Current National and International Studies*

mindLAMP already has a track record of successful uses in many diverse clinical research settings. The app has received local IRB approval and has been deployed not only in its Harvard/Beth Israel Deaconess home site but also sites around the world.[^7–9^](https://paperpile.com/c/zes0fh/0AuPn+RWOJY+4vp8R) The ability of each of these sites to use mindLAMP reflects its flexible nature, practical value, alignment with privacy/security regulations

*6-Clinical Care*

mindLAMP is unique in that it is more than a research tool and is already deployed in clinical care.[^10^](https://paperpile.com/c/zes0fh/mjIJ1) These clinical use cases for mindLAMP offer a direct pathway for translational research to have an immediate impact on care. The clinical use of mindLAMP also informs its development and ensures that research and clinical needs find synergy. mindLAMP can also support learning by offering on-demand psychoeducation, and management through just-in-time adaptive interventions[^11^](https://paperpile.com/c/zes0fh/t1cPO) (e.g., elevated anxiety scores trigger a mindfulness activity in the app). There is also a patient portal to review all data captured and the ability to chat within the dashboard. These features can be enabled or disabled depending on the clinical or research needs. Of note, the clinical use of mindLAMP is not part of AMP SZ but highlights immediate relevance of findings.

Unique to this study, the mindLAMP app is available in nine languages (English, Spanish, French, Italian, German, Korean, Traditional and Simplified Chinese, and Danish), offers interactive data sharing possibilities for the users, and has custom security features and data pipelines to enable the first ever sharing of this type of data with the National Data Archive. All data is automatically and securely transferred to study servers to enable remote research.

Cognition

Each Wechsler assessment instrument contains the same subtests used in this study, ensuring construct validity across instruments. While the ideal approach would be to administer the same battery to all sites (i.e., English speaking and non-English speaking), the WASI-II was chosen for use where possible for several reasons: 1) a large majority of our sites speak English; 2) unlike the WAIS-IV and the WISC-V, which are normed as comprehensive measures of intellect, the WASI-II is normed as a brief measure of intellect that is both more reliable and valid psychometrically for this purpose. As the ‘gold standard’ in this case, we will also compare the reliability and validity of the WAIS-IV / WISC-IV scores with the WASI-II scores; 3) The WASI-II is normed for use in participants between the ages of 6 and 90, providing coverage of the age range of the study sample, while other sites will need to use either the WAIS-IV or the WISC-IV depending on participant’s age; 4) The WASI-II is a considerably more economical purchase than the WAIS-IV/WISC-V batteries.

Electrophysiology

To minimize site differences, a detailed standard operating procedure document was created, along with training videos, that were accessible from a website dedicated to the project. Site EEG set-ups were photographed and reviewed by the EEG leadership team to ensure optimal room configurations. Site research staff submitted practice EEG acquisition sessions acquired from lab volunteers. These sessions were reviewed and had to meet minimum standards of data quality and procedural accuracy for the staff member to be formally certified. Only certified staff were permitted to acquire EEG data. As part of the DPACC, a web application^14^ was built to display raw and processed data, as well as quality control metrics, for each EEG session. Each EEG was scored on a scale of 1-4, 1 being poor/unusable and 4 being excellent. Data uploaded by sites were automatically detected and processed, and reports of data were available to be viewed by sites on a dedicated web-based dashboard within 1-3 days of upload. Near real-time detection of data problems led to rapid feedback and, in some cases, return of the participant to repeat the EEG session. EEG session reports were also reviewed and discussed during weekly video calls attended by all sites, creating a “virtual” community of EEG acquisition staff and investigators collaborating on ways to optimize data quality and uniformity across sites while collaboratively working to troubleshoot problems or address challenges.

Neuroimaging

Custom automatized data flow processes were designed to transfer imaging data from individual sites to the research network hubs and to the DPACC. Data are automatically deidentified upon intake at the research hubs and upon arrival at the DPACC go through automated QC checks that validate the consistency of the data and adherence to the specified imaging protocol. These automated checks include comparison of numerous imaging parameters in a given session to the expected protocol parameters for that site, checks of image matrix size and resolution, slice selection consistency, and shim consistency, testing for DICOM bit depth (16- versus 12-bit), and tests for DICOM format. Following QC, the data will be mathematically harmonized to eliminate residual site effects (e.g., the diffusion MRI data will be harmonized using rotation-invariant spherical harmonics features[^12^](https://paperpile.com/c/zes0fh/fa1m4)), and will then be processed using robust analysis streams such as those developed for the HCP studies[^13–16^](https://paperpile.com/c/zes0fh/1puTw+3Uu5X+rWaTO+G09MD), and those used in the PNLpipe package[^17^](https://paperpile.com/c/zes0fh/g0jVq). In addition to structural measures conventionally generated from the HCP pipeline outputs (e.g., cortical thickness, volume, and T1w/T2w myelin maps [^18^](https://paperpile.com/c/zes0fh/thGIM)), we will generate functional connectomes and diffusion microstructural measures. Advanced measures such as Free-water [^19^](https://paperpile.com/c/zes0fh/Jggm4), kurtosis [^20^](https://paperpile.com/c/zes0fh/nvptW), neurite orientation dispersion and density imaging (NODDI) [^21^](https://paperpile.com/c/zes0fh/NVKZY), shape characteristics of tractography[^22^](https://paperpile.com/c/zes0fh/niMFK), and graph theory indices from the functional data [^23^](https://paperpile.com/c/zes0fh/yk2ur) will be considered as possible “image derived phenotypes” by the Neuroimaging workgroup and the Data Analytic Strategy workgroup.

Finally, although neurochemical or molecular imaging were considered, the inclusion of magnetic resonance spectroscopy (MRS) would have added considerable complexity to this multi-vendor, multi-platform study. For example, acquisition of quality MRS data requires advanced shimming and careful placement of anatomical regions of interest. Additionally, acquisition time was not available to add another modality to the imaging protocol given the need for the protocol to be completed in a 60 minute scheduled imaging slot. As such, neurochemical or molecular imaging were omitted from AMP SCZ.

Spoken language and facial expression sampling.

Language, shaped by cognitive processes, offers considerable potential in forecasting psychosis. We'll employ three methods to gather language samples:

1. Open-ended Interviews: Lasting approximately 20 minutes, these non-directive sessions invite participants to talk freely about their lives. Commencing with the prompt, “I'd love to understand your life better. How have things been lately?” these interviews shed light on the participants' prevailing concerns. Due to their unstructured nature, they are likely to offer insights into conceptual coherence more effectively than structured interviews. Moreover, the non-directive approach arguably captures conversational dynamics better than semi-structured interviews focusing on symptoms.

2. PSYCHS Interviews: These semi-structured sessions, carried out multiple times during the study, aim to gauge the risk of psychosis. Interviewers will specifically inquire about certain symptoms, facilitating a comparison between the content discussed and the clinical evaluations produced by the interviewer.

3. Daily Diaries: Participants will chronicle daily reflections, typically lasting around 3 minutes, via their smart devices or the devices provided. Managed through the mindLAMP app, these entries offer real-time insights into participants' sentiments and contemplations outside the clinic at a moment of their choosing. Given the private setting, it's plausible that participants will be more candid, with fewer social pressures influencing their responses.

For the open-ended interviews, we'll use Zoom, a platform that supports both video and audio capture. Crucially, Zoom allows the disabling of noise cancellation and provides speaker diarization, segregating each speaker's audio — essential for detailed speech analysis. PSYCHS interviews will either be recorded on Zoom or with a digital recorder capable of saving audio in the .WAV format, which maintains high audio quality for acoustic analysis. Meanwhile, the daily diary samples will be stored as .m4a files, suitable for voice transcription.

However, communication extends beyond mere words. Facial expressions and distinctive vocal characteristics are also pivotal. Therefore, we will assess facial action units and speech acoustics. Facial action units, which denote individual muscle movements forming facial expressions, will be gauged using MediaPipe v0.9.3.0 at a 20ms sampling rate. We'll use Praat v6.3.1077 and openSMILE v3.0 to measure acoustic properties, extracting attributes such as loudness, spectral energy, and speech pace.

A cornerstone of our analysis will be juxtaposing speech transcriptions with facial and vocal attributes. Artificial Intelligence, specifically Large Language Models (LLMs), will assist in deducing emotions like anger or confusion, cognitive states like confusion and certainty, and cognitive attributes such as working memory span and memory encoding accuracy. Once these emotional and cognitive features are extracted, they can be aligned with corresponding facial and vocal expressions, revealing the intertwined semantics of face and voice.

While current machine-based transcription is evolving, human transcription remains the gold standard for accuracy, especially in recognizing potential confidentiality risks. All Protected Health Information (PHI) and Personally Identifiable Information (PII) will be diligently redacted to safeguard participant confidentiality. As machine transcription and PII/PHI detection technologies advance, we remain open to their adoption once successfully validated against human transcription.

Sample size and power

We used a recently-described method developed by Riley et al.[^24^](https://paperpile.com/c/zes0fh/rlFNM) for developing a clinical prediction model using a traditional likelihood-based approach (e.g., logistic regression) to determine our required sample size. Sample sizes were derived for the scenarios of having low, medium or high prediction performance and also for the number of parameters in the model being 10, 20, 30, 40 or 50. The expected conversion rate used was 15% over 1 year. Using the R package pmsampsize (https://cran.r-project.org/web/packages/pmsampsize/index.html),[^24–26^](https://paperpile.com/c/zes0fh/rlFNM+K3yjj+chsMl) we estimated required sample sizes for binary (i.e., conversion versus non-conversion) and survival (accounting for time to conversion and censoring) outcomes, using assumed low/medium/high *R^2^* values of 0.25/0.40/0.55 for the binary outcome model, and 0.20/0.30/0.40 for the survival outcome model.

For the binary outcome model, the command was:

*pmsampsize(type=”b”,rsquared=<R2>,parameters=<#params>,shrinkage=0.9,
prevalance=0.15)*

For the survival outcome model, the command was:

*pmsampsize(type=”s”,rsquared=<R2>,parameters=<#params>,shrinkage=0.9,
rate-0.075,timepoint=2,meanfup=1)*

where *<R2>* and *<#params>* represent one of the values specified above.

Note that for the survival outcome model we assumed that conversion occurred over a 2 year period, so we used a rate value of 0.075 (= prevalence / 2).

For binary or survival (time-to-event) outcomes, there are three criteria for determining sample size:

1. small overfitting defined by an expected shrinkage of predictor effects by 10% or less,

2. small absolute difference of 0.05 in the model's apparent and adjusted Nagelkerke's R-squared value[^27^](https://paperpile.com/c/zes0fh/qxWqy), and

3. precise estimation (within +/- 0.05) of the average outcome risk in the population for a key timepoint of interest for prediction.

Each criterion may require a different sample size and the chosen sample size is the largest of the three. For this study, when the outcome is considered as binary, the criterion with the largest required sample size is criterion 1 for low predictive performance and 2 for medium and high performance. When the outcome is considered as survival, the largest required sample size corresponds to both criteria 2 and 3 for all levels of predictive performance.

Table S1 shows the resulting sample sizes for binary outcome and Table S2 for survival outcome.

Table S1. Minimum sample sizes for binary outcome.

|  | | Number of parameters in model | | | | |
| --- | --- | --- | --- | --- | --- | --- |
|  |  | 10 | 20 | 30 | 40 | 50 |
| Predictive performance | low | 308 | 615 | 922 | 1230 | 1537 |
|  | medium | 267 | 534 | 800 | 1067 | 1333 |
|  | high | 237 | 473 | 709 | 946 | 1182 |

Table S2. Minimum sample sizes for survival outcome.

|  | | Number of parameters in model | | | | |
| --- | --- | --- | --- | --- | --- | --- |
|  |  | 10 | 20 | 30 | 40 | 50 |
| Predictive performance | low | 422 | 843 | 1265 | 1686 | 2108 |
|  | medium | 398 | 796 | 1193 | 1591 | 1989 |
|  | high | 366 | 732 | 1098 | 1464 | 1830 |

Assuming a conversion rate of 15%, and a maximum of 30 parameters included in the predictive model, the minimum sample size for a model is estimated to be between 1100 and 1300 for a survival outcome. A maximum of 30 parameters was chosen as a balance between inclusion of a sufficient number of multimodal predictors and not being overly cumbersome in practice. Our projected sample size of 1,977 CHR young people is therefore adequate for developing a prediction model with ~30 parameters that has high predictive performance, while still allowing a subset of the data to be ‘held back’ for internal validation purposes.

Table S3. AMP SCZ Schedule of Assessments

| **Domain** | **Instrument/**  **Specimen** | **Screening** | **M0** | **M1** | **M2** | **M3** | **M4** | **M5** | **M6** | **M7** | **M8** | **M9** | **M10** | **M11** | **M12** | **M18** | **M24** | **Conversion** |
| --- | --- | --- | --- | --- | --- | --- | --- | --- | --- | --- | --- | --- | --- | --- | --- | --- | --- | --- |
| **INFORMED CONSENT** | | | | | | | | | | | | | | | | | | |
|  | Informed consent | ✔C |  |  |  |  |  |  |  |  |  |  |  |  |  |  |  |  |
| **CLINICAL** | | | | | | | | | | | | | | | | | | |
| **Inclusion/Exclusion criteria** | PSYCHS/SOFAS/SCID5-PD-Schizotypal /FIGS (abbreviated version)/ TBI/medication use (PharmaTreat) | ✔C |  |  |  |  |  |  |  |  |  |  |  |  | C |  | C |  |
| **Health Conditions (Medical/Psychiatric History)** | Health and Medical Conditions Questionnaires | ✔C | ✔ | ✔ | ✔C | ✔ | ✔ | ✔ | ✔ | ✔ | ✔ | ✔ | ✔ | ✔ | ✔C | ✔ | ✔C |  |
| **Health Conditions (Genetics and Fluid Biomarkers)** | Health and Medical Conditions Questionnaires | ✔C |  |  |  |  |  |  |  |  |  |  |  |  |  |  |  |  |
| **Demographics** | **Demographics** |  | ✔C |  |  |  |  |  |  |  |  |  |  |  |  |  |  |  |
| **Premorbid functioning** | PAS |  |  | ✔ |  |  |  |  |  |  |  |  |  |  |  |  |  |  |
| **Adverse events** | **Adverse Events** |  | ✔C | ✔C | ✔C | ✔C | ✔C | ✔C | ✔C | ✔C | ✔C | ✔C | ✔C | ✔C | ✔C | ✔C | ✔C |  |
| **Attenuated psychotic symptoms, associated distress, and conversion to psychosis** | PSYCHS |  | ✔C | ✔ | ✔C | ✔ |  |  | ✔ |  |  |  |  |  | ✔ C | ✔ | ✔ C | ✔ |
| **General**  **psychopathology** | BPRS |  | ✔C | ✔ | ✔C | ✔ | ✔ | ✔ | ✔ | ✔ | ✔ | ✔ | ✔ | ✔ | ✔ | ✔ | ✔ | ✔ |
| **Depression** | CDSS |  | ✔C | ✔ | ✔C | ✔ |  |  | ✔ |  |  |  |  |  | ✔ | ✔ | ✔ | ✔ |
| **Anxiety** | OASIS |  | ✔C | ✔ | ✔C | ✔ |  |  | ✔ |  |  |  |  |  | ✔ | ✔ | ✔ | ✔ |
| **Suicidality** | CSSRS |  | ✔C |  | ✔C |  |  |  | ✔ |  |  |  |  |  | ✔ | ✔ | ✔ | ✔ |
| **Sleep disturbance** | PROMIS-SD |  | ✔C |  | ✔C |  |  |  | ✔ |  |  |  |  |  | ✔ | ✔ | ✔ | ✔ |
| **Substance use** | ASSIST |  | ✔C |  | ✔✔ |  |  |  | ✔ |  |  |  |  |  | ✔ | ✔ | ✔ | ✔ |
| **DSM diagnoses** | SCID-5-RVs (Psychosis, Mood, Substance Abuse) |  | ✔C |  |  |  |  |  |  |  |  |  |  |  | ✔C |  | ✔C | ✔ |
| **Patient global impression of severity** | PGI-S |  | ✔C | ✔ | ✔C | ✔ |  |  | ✔ |  |  |  |  |  | ✔ | ✔ | ✔ | ✔ |
| **Psychosocial functioning** | SOFAS, GF: Social, GF: Role | ✔C | ✔C | ✔ | ✔C | ✔ |  |  | ✔ |  |  |  |  |  | ✔ C | ✔ | ✔ C | ✔ |
| **Perceived Stress** | PSS |  | ✔C | ✔ | ✔C | ✔ |  |  | ✔ |  |  |  |  |  | ✔ | ✔ | ✔ | ✔ |
| **Perceived Discrimination** | PDQ |  | ✔C |  |  |  |  |  |  |  |  |  |  |  |  |  |  |  |
| **Pubertal development** | PDS |  | ✔C |  |  |  |  |  |  |  |  |  |  |  |  |  |  |  |
| **Psychosis Polyrisk Score** | PPS |  | ✔C |  |  |  |  |  |  |  |  |  |  |  |  |  |  |  |
| **DIGITAL MOMENTARY ASSESSMENTS** | | | | | | | | | | | | | | | | | | |
| **Daily changes in mental state and context** | EMA (MindLAMP) |  | ✔C | ✔ C | ✔ C | ✔ C | ✔ C | ✔ C | ✔ C | ✔ C | ✔ C | ✔ C | ✔ C | ✔ C | ✔ C |  |  |  |
| **Physical activity, sleep-wake cycles, travel patterns** | Passive sensing (actigraphy, geolocation; Axivity) |  | ✔ C | ✔ C | ✔ C | ✔ C | ✔ C | ✔ C | ✔ C | ✔ C | ✔ C | ✔ C | ✔ C | ✔ C | ✔ C |  |  |  |
| **COGNITION** | | | | | | | | | | | | | | | | | | |
| **Premorbid IQ** | WRAT5 Word Reading |  | ✔C |  |  |  |  |  |  |  |  |  |  |  |  |  |  |  |
| **Current IQ** | WASI-II - 2-subtest version (Vocab & MR) |  | ✔C |  |  |  |  |  |  |  |  |  |  |  |  |  | ✔ |  |
| **Processing speed** | Digit-Symbol Substitution Test |  | ✔C |  | ✔C |  |  |  | ✔ |  |  |  |  |  | ✔ |  | ✔ |  |
| **Attention** | Short Penn Continuous Performance Test |  | ✔C |  | ✔C |  |  |  | ✔ |  |  |  |  |  | ✔ |  | ✔ |  |
| **Working memory** | Short Fractal N-Back Test |  | ✔C |  | ✔C |  |  |  | ✔ |  |  |  |  |  | ✔ |  | ✔ |  |
| **Relational Memory** | Digit-Symbol Test |  | ✔C |  | ✔C |  |  |  | ✔ |  |  |  |  |  | ✔ |  | ✔ |  |
| **Visual memory** | Short Visual Object Learning Test |  | ✔C |  | ✔C |  |  |  | ✔ |  |  |  |  |  | ✔ |  | ✔ |  |
| **Verbal learning** | Short Penn List Learning Test |  | ✔C |  | ✔C |  |  |  | ✔ |  |  |  |  |  | ✔ |  | ✔ |  |
| **Emotion recognition** | Penn Emotion Recognition Test |  | ✔C |  | ✔C |  |  |  | ✔ |  |  |  |  |  | ✔ |  | ✔ |  |
| **Motor** | Short Computerised Finger Tapping Test |  | ✔C |  | ✔C |  |  |  | ✔ |  |  |  |  |  | ✔ |  | ✔ |  |
| **Sensorimotor speed** | Motor Praxis Test |  | ✔C |  | ✔C |  |  |  | ✔ |  |  |  |  |  | ✔ |  | ✔ |  |
| **ELECTROPHYSIOLOGY (EEG)** | | | | | | | | | | | | | | | | | | |
| **Mismatch negativity concurrent with Visual oddball** | Mismatch negativity and visual target/novelty P300 |  | ✔C |  | ✔C |  |  |  |  |  |  |  |  |  |  |  |  |  |
| **Auditory oddball** | Auditory target/novelty P300  Auditory target/novel alpha desynchronisation |  | ✔C |  | ✔C |  |  |  |  |  |  |  |  |  |  |  |  |  |
| **40 HZ auditory steady state response** | 40Hz power and inter-trial phase coherence, baseline inter-stimulus interval gamma power |  | ✔C |  | ✔C |  |  |  |  |  |  |  |  |  |  |  |  |  |
| **Resting state EEG (eyes open/closed)** | Power spectra  1/f slope |  | ✔C |  | ✔C |  |  |  |  |  |  |  |  |  |  |  |  |  |
| **NEUROIMAGING (MRI)** | | | | | | | | | | | | | | | | | | |
| **Structural/functional**  **(incl resting state)** | T1, T2, diffusion MRI, resting state functional MRI |  | ✔C |  | ✔C |  |  |  |  |  |  |  |  |  |  |  |  |  |
| **GENETICS & FLUID BIOMARKERS** | | | | | | | | | | | | | | | | | | |
| **Vital signs** | Elevated body mass index, blood pressure, temperature |  | ✔C |  | ✔C |  |  |  |  |  |  |  |  |  |  |  |  |  |
| **Current health status and activity** | Current illnesses and recent activity |  | ✔C |  | ✔C |  |  |  |  |  |  |  |  |  |  |  |  |  |
| **Elevations in white blood cells** | Blood sample – CBC with differential |  | ✔C |  | ✔C |  |  |  |  |  |  |  |  |  |  |  |  |  |
| **Immune system, coagulation system, complement system, and oxidative stress** | Blood sample – plasma, serum (multiplex, ELISA/mass spectrometry) |  | ✔C |  | ✔C |  |  |  |  |  |  |  |  |  |  |  |  |  |
| **DNA isolated for microarray/low-pass sequencing** | Blood sample – buffy coat |  | ✔C |  | ✔C |  |  |  |  |  |  |  |  |  |  |  |  |  |
| **Functional assays for redox dysregulation and cell membranes for lipids (e.g. DHA/EPA/AA)** | Whole blood sample (functional assays, mass spectrometry, gas chromatography) |  | ✔C |  | ✔C |  |  |  |  |  |  |  |  |  |  |  |  |  |
| **Cortisol** | Saliva Collection (ELISA) |  | ✔C |  | ✔C |  |  |  |  |  |  |  |  |  |  |  |  |  |
| **SPEECH and FACIAL EXPRESSION** | | | | | | | | | | | | | | | | | | |
| **Language content and structure**  **Speech acoustics** | Free speech recording (Zoom audio) |  | ✔C |  | ✔C |  |  |  |  |  |  |  |  |  |  |  |  |  |
|  | PSYCHS interview recording |  | ✔C | ✔C | ✔C | ✔C |  |  | ✔C |  |  |  |  |  | ✔C | ✔C | ✔C | ✔ |
|  | Audio diaries recorded via smartphone as component of EMA (2 mins daily) |  | ✔ C | ✔ C | ✔ C | ✔ C | ✔ C | ✔ C | ✔ C | ✔ C | ✔ C | ✔ C | ✔ C | ✔ C | ✔ C |  |  |  |
| **Facial expression** | Free speech recording (Zoom video) |  | ✔C |  | ✔C |  |  |  |  |  |  |  |  |  |  |  |  |  |
| **TREATMENT AND HEALTH SERVICE UTILISATION** | | | | | | | | | | | | | | | | | | |
|  | Psychosocial/  pharmacological treatment/service use |  | ✔C | ✔ | ✔C | ✔ |  |  | ✔ |  |  |  |  |  | ✔C | ✔ | ✔C |  |

✔ = CHR participants; C = HC participants (5 of the 15 HC at each site and 15 of the 100 HC in Melbourne will receive repeat biomarker assessments)

*Abbreviations:* PSYCHS, Positive Symptoms and Diagnostic Criteria for the CAARMS Harmonized with the SIPS; SOFAS, Social and Occupational Functioning Scale; SCID5-PD, Structured Clinical Interview for DSM-5 - Personality Disorders; FIGS, Family Interview for Genetic Studies; TBI, Traumatic Brain Injury; PAS, Premorbid Adjustment Scale; BPRS, Brief Psychiatric Rating Scale; CDSS, Calgary Depression Scale for Schizophrenia; OASIS, Overall Anxiety Severity And Impairment Scale; CSSRS, Columbia Suicide Severity Rating Scale; PROMIS-SD, Patient Reported Outcomes Measurement Information System-Sleep Disturbance; ASSIST, Alcohol, Smoking and Substance Involvement Screening Test; SCID-5-RVs, Structured Clinical Interview for DSM-5 - Research Version; PGI-S, Patient Global Impression of Severity; GF, Global Functioning; PSS, Perceived Stress Scale; PDQ, Perceived Discrimination Questionnaire; PDS, Pubertal Development Scale; PPS, Psychosis Polyrisk Score; EMA, Ecological Momentary Assessment; WRAT-5, Wide Range Achievement Test-Fifth Edition; WASI-II, Wechsler Abbreviated Scale of Intelligence, Second Edition; CBC, complete blood count.

**References**

1. [Carney R, Yung AR, Amminger GP, et al. Substance use in youth at risk for psychosis. *Schizophr Res*. 2017;181:23-29.](http://paperpile.com/b/zes0fh/0mhCj)

2. [Cohen A, Naslund JA, Chang S, et al. Relapse prediction in schizophrenia with smartphone digital phenotyping during COVID-19: a prospective, three-site, two-country, longitudinal study. *Schizophrenia (Heidelb)*. 2023;9(1):6.](http://paperpile.com/b/zes0fh/IUEdl)

3. [Henson P, Barnett I, Keshavan M, Torous J. Towards clinically actionable digital phenotyping targets in schizophrenia. *NPJ Schizophr*. 2020;6(1):13.](http://paperpile.com/b/zes0fh/ARrT5)

4. [Henson P, Torous J. Feasibility and correlations of smartphone meta-data toward dynamic understanding of depression and suicide risk in schizophrenia. *Int J Methods Psychiatr Res*. 2020;29(2):e1825.](http://paperpile.com/b/zes0fh/56L7d)

5. [Shvetz C, Gu F, Drodge J, Torous J, Guimond S. Validation of an ecological momentary assessment to measure processing speed and executive function in schizophrenia. *NPJ Schizophr*. 2021;7(1):64.](http://paperpile.com/b/zes0fh/3xDKp)

6. [Carsten Langholm , Tobias Kowatsch , Sandra Bucci, Andrea Cipriani, John Torous. Exploring the potential of Apple SensorKit and digital phenotyping data as new digital biomarkers for mental health research. *Digital Biomakers*. Published online 2023.](http://paperpile.com/b/zes0fh/Ft7mr)

7. [Rodriguez-Villa E, Mehta UM, Naslund J, et al. Smartphone Health Assessment for Relapse Prevention (SHARP): a digital solution toward global mental health. *BJPsych Open*. 2021;7(1):e29.](http://paperpile.com/b/zes0fh/0AuPn)

8. [Li H, Yang S, Chi H, et al. Enhancing attention and memory of individuals at clinical high risk for psychosis with mHealth technology. *Asian J Psychiatr*. 2021;58:102587.](http://paperpile.com/b/zes0fh/RWOJY)

9. [Bilden R, Torous J. Global Collaboration Around Digital Mental Health: The LAMP Consortium. *J Technol Behav Sci*. 2022;7(2):227-233.](http://paperpile.com/b/zes0fh/4vp8R)

10. [Rauseo-Ricupero N, Henson P, Agate-Mays M, Torous J. Case studies from the digital clinic: integrating digital phenotyping and clinical practice into today’s world. *Int Rev Psychiatry*. 2021;33(4):394-403.](http://paperpile.com/b/zes0fh/mjIJ1)

11. [Vaidyam A, Halamka J, Torous J. Actionable digital phenotyping: a framework for the delivery of just-in-time and longitudinal interventions in clinical healthcare. *Mhealth*. 2019;5:25.](http://paperpile.com/b/zes0fh/t1cPO)

12. [Chen AA, Beer JC, Tustison NJ, et al. Mitigating site effects in covariance for machine learning in neuroimaging data. *Hum Brain Mapp*. 2022;43(4):1179-1195.](http://paperpile.com/b/zes0fh/fa1m4)

13. [Van Essen DC, Smith SM, Barch DM, et al. The WU-Minn Human Connectome Project: an overview. *Neuroimage*. 2013;80:62-79.](http://paperpile.com/b/zes0fh/1puTw)

14. [Sotiropoulos SN, Jbabdi S, Xu J, et al. Advances in diffusion MRI acquisition and processing in the Human Connectome Project. *Neuroimage*. 2013;80:125-143.](http://paperpile.com/b/zes0fh/3Uu5X)

15. [Sporns O. The human connectome: origins and challenges. *Neuroimage*. 2013;80:53-61.](http://paperpile.com/b/zes0fh/rWaTO)

16. [Glasser MF, Sotiropoulos SN, Wilson JA, et al. The minimal preprocessing pipelines for the Human Connectome Project. *Neuroimage*. 2013;80:105-124.](http://paperpile.com/b/zes0fh/G09MD)

17. [reckbo, Billah T, Norton I. *Pnlbwh/pnlpipe: Easy Install and Multiprocessing*. Zenodo; 2019. doi:](http://paperpile.com/b/zes0fh/g0jVq)[10.5281/ZENODO.2584271](http://dx.doi.org/10.5281/ZENODO.2584271)

18. [Glasser MF, Van Essen DC. Mapping human cortical areas in vivo based on myelin content as revealed by T1- and T2-weighted MRI. *J Neurosci*. 2011;31(32):11597-11616.](http://paperpile.com/b/zes0fh/thGIM)

19. [Pasternak O, Sochen N, Gur Y, Intrator N, Assaf Y. Free water elimination and mapping from diffusion MRI. *Magn Reson Med*. 2009;62(3):717-730.](http://paperpile.com/b/zes0fh/Jggm4)

20. [Zhang F, Ning L, O’Donnell LJ, Pasternak O. MK-curve - Characterizing the relation between mean kurtosis and alterations in the diffusion MRI signal. *Neuroimage*. 2019;196:68-80.](http://paperpile.com/b/zes0fh/nvptW)

21. [Zhang H, Schneider T, Wheeler-Kingshott CA, Alexander DC. NODDI: practical in vivo neurite orientation dispersion and density imaging of the human brain. *Neuroimage*. 2012;61(4):1000-1016.](http://paperpile.com/b/zes0fh/NVKZY)

22. [Yeh FC. Shape analysis of the human association pathways. *Neuroimage*. 2020;223:117329.](http://paperpile.com/b/zes0fh/niMFK)

23. [Wang J, Zuo X, He Y. Graph-based network analysis of resting-state functional MRI. *Front Syst Neurosci*. 2010;4:16.](http://paperpile.com/b/zes0fh/yk2ur)

24. [Riley RD, Ensor J, Snell KIE, et al. Calculating the sample size required for developing a clinical prediction model. *BMJ*. 2020;368:m441.](http://paperpile.com/b/zes0fh/rlFNM)

25. [Riley RD, Snell KIE, Ensor J, et al. Minimum sample size for developing a multivariable prediction model: Part I - Continuous outcomes. *Stat Med*. 2019;38(7):1262-1275.](http://paperpile.com/b/zes0fh/K3yjj)

26. [Riley RD, Snell KI, Ensor J, et al. Minimum sample size for developing a multivariable prediction model: PART II - binary and time-to-event outcomes. *Stat Med*. 2019;38(7):1276-1296.](http://paperpile.com/b/zes0fh/chsMl)

27. [Nagelkerke NJD. A note on a general definition of the coefficient of determination. *Biometrika*. 1991;78(3):691-692.](http://paperpile.com/b/zes0fh/qxWqy)
